# Molecular Portraits of Early Rheumatoid Arthritis Identify Clinical and Treatment Response Phenotypes

**DOI:** 10.1016/j.celrep.2019.07.091

**Published:** 2019-08-27

**Authors:** Myles J. Lewis, Michael R. Barnes, Kevin Blighe, Katriona Goldmann, Sharmila Rana, Jason A. Hackney, Nandhini Ramamoorthi, Christopher R. John, David S. Watson, Sarah K. Kummerfeld, Rebecca Hands, Sudeh Riahi, Vidalba Rocher-Ros, Felice Rivellese, Frances Humby, Stephen Kelly, Michele Bombardieri, Nora Ng, Maria DiCicco, Désirée van der Heijde, Robert Landewé, Annette van der Helm-van Mil, Alberto Cauli, Iain B. McInnes, Christopher D. Buckley, Ernest Choy, Peter C. Taylor, Michael J. Townsend, Costantino Pitzalis

**Affiliations:** 1 Centre for Experimental Medicine and Rheumatology, William Harvey Research Institute, Barts and The London School of Medicine and Dentistry, Queen Mary University of London, Charterhouse Square, London EC1M 6BQ, UK; 2 Centre for Translational Bioinformatics, William Harvey Research Institute, Barts and The London School of Medicine and Dentistry, Queen Mary University of London, Charterhouse Square, London EC1M 6BQ, UK; 3 Alan Turing Institute, British Library, London NW1 2DB, UK; 4 Bioinformatics and Computational Biology, Genentech Research & Early Development, 1 DNA Way, South San Francisco, CA 94080, USA; 5 Biomarker Discovery OMNI, Genentech Research & Early Development, 1 DNA Way, South San Francisco, CA 94080, USA; 6 Oxford Internet Institute, University of Oxford, Oxford OX1 3JS, UK; 7 Department of Rheumatology, Leiden University Medical Center, the Netherlands; 8 Department of Clinical Immunology & Rheumatology, Amsterdam Rheumatology & Immunology Center, Amsterdam, the Netherlands; 9 Rheumatology Unit, Department of Medical Sciences, Policlinico of the University of Cagliari, Cagliari, Italy; 10 Institute of Infection, Immunity and Inflammation, University of Glasgow, Glasgow G12 8TA, UK; 11 Rheumatology Research Group, Institute of Inflammation and Ageing (IIA), University of Birmingham, Birmingham B15 2WB, UK; 12 Nuffield Department of Orthopaedics, Rheumatology and Musculoskeletal Sciences and the Kennedy Institute of Rheumatology, University of Oxford, Oxford, UK; 13 Institute of Infection and Immunity, Cardiff University School of Medicine, Cardiff CF14 4XN, UK

**Keywords:** rheumatoid arthritis, RNA sequencing, personalized medicine, synovial biopsy, ectopic lymphoid structures, lymphoid neogenesis, transcriptomics, Pathobiology of Early Arthritis Cohort study, PEAC

## Abstract

There is a current imperative to unravel the hierarchy of molecular pathways that drive the transition of early to established disease in rheumatoid arthritis (RA). Herein, we report a comprehensive RNA sequencing analysis of the molecular pathways that drive early RA progression in the disease tissue (synovium), comparing matched peripheral blood RNA-seq in a large cohort of early treatment-naive patients, namely, the Pathobiology of Early Arthritis Cohort (PEAC). We developed a data exploration website (https://peac.hpc.qmul.ac.uk/) to dissect gene signatures across synovial and blood compartments, integrated with deep phenotypic profiling. We identified transcriptional subgroups in synovium linked to three distinct pathotypes: fibroblastic pauci-immune pathotype, macrophage-rich diffuse-myeloid pathotype, and a lympho-myeloid pathotype characterized by infiltration of lymphocytes and myeloid cells. This is suggestive of divergent pathogenic pathways or activation disease states. Pro-myeloid inflammatory synovial gene signatures correlated with clinical response to initial drug therapy, whereas plasma cell genes identified a poor prognosis subgroup with progressive structural damage.

## Highlights

• Deep phenotyping and RNA-seq of early rheumatoid arthritis individuals pre-treatment
• Synovial plasma cell gene expression predicts future progressive joint damage on X-ray
• Blood interferon gene signature associates with synovial B and plasma cell infiltration
• Interactive website enables RNA-seq and clinical data to be fully explored

## Graphical Abstract

**Figure:**
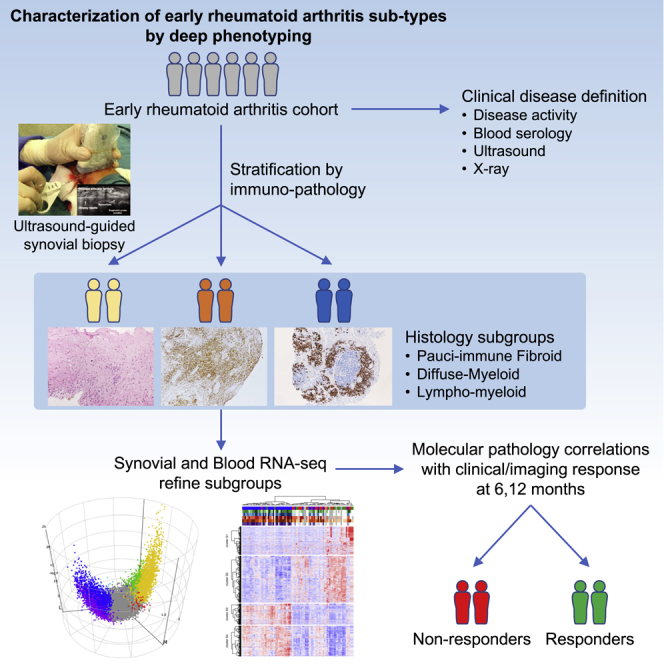

## Teaser

Lewis et al. use histology and RNA-seq of synovial biopsies from a cohort of early rheumatoid arthritis individuals to identify three histological pathotypes and reveal gene modules associated with disease severity and clinical response.

## Introduction

The genetic architecture underlying susceptibility to rheumatoid arthritis (RA) (Eyre et al., 2017) and its interaction with environmental and epigenetic factors have been characterized with increasing depth. Although these predisposing factors initiate the start of RA, the subsequent aberrant biological processes and sequence of events, which drive the transition from systemic autoimmunity to joint inflammation, and from early to established disease, culminating in the development of synovitis and articular destruction, are less clear (Firestein and McInnes, 2017). Established RA displays clinical heterogeneity as demonstrated by variable prognosis, unpredictable propensity to rapid progression to structural damage, and inconsistent response to therapy. Although RA treatment has been revolutionized by biologic and synthetic therapies targeting specific immune-mediated pathways, a significant number of patients fail to respond to current medications with only 20%–30% reaching low disease activity status (as measured by 70% improvement in American College of Rheumatology [ACR] response criteria). Notably, treatment failure rates remain uniformly similar, regardless of the drug mechanism of action. The mechanistic reasons for such similar failure rates remain largely unknown, but the wide cellular and molecular variation described in the synovial tissue of patients with long-standing RA are likely to play a role in the variable treatment response and heterogeneous clinical evolution (Pitzalis et al., 2013).

Although a number of studies have examined synovial tissue gene expression (Badot et al., 2009, Dennis et al., 2014, Lindberg et al., 2010, Timmer et al., 2007), few studies have focused on gene expression in early, treatment-naive RA (De Groof et al., 2016). Many of these studies have been performed in established or late stage disease and with a sampling bias due to major representation of large joints, whereas only a handful of studies have included small joints (Pitzalis et al., 2013). Furthermore, very few studies have to date reported a systematic molecular characterization by RNA sequencing of the synovial tissue (Mandelin et al., 2018, Orange et al., 2018) but none in early treatment-naive patients. Until now it has remained unknown whether specific histological and transcriptomic findings represent an evolutionary response to long-standing joint inflammation following multiple immune-modulatory therapies or embody distinct, essential RA pathogenetic mechanisms from disease onset. In addition, although microarray-based gene expression analyses have been performed in RA peripheral blood (Smith et al., 2013), no studies have examined coordinate gene expression changes at the RNA sequencing level in blood and synovium from the same patients.

Here, we characterize at disease presentation early, pre-treatment RA (mean 5.6-month symptom duration), using comprehensive RNA sequencing (RNA-seq) analysis of synovial biopsies and blood from the largest biopsy-driven cohort of treatment-naive patients: the Pathobiology of Early Arthritis Cohort (PEAC). We combined RNA-seq with detailed synovial histology and correlated these molecular signatures with clinical and imaging phenotype data at disease presentation. We show that the spectrum of the synovial immune response is diverse and associated with differential blood immune signals. We identify transcriptional endotypes in the synovium linked to three distinct pathotypes: fibroblastic-rich pauci-immune pathotype, myeloid- or macrophage-rich pathotype, and lymphoid-rich pathotype with high plasma cell accumulation. This study yields major insights into pathogenic pathways in RA synovium and the links between local synovium and systemic immune responses in the blood and demonstrates that synovial pathotype signatures are associated with diverse disease activity or severity and structural damage at baseline and response to disease-modifying anti-rheumatic drug (DMARD) therapy.

## Results

### Identification of Distinct Histological Pathotypes in Treatment-Naive Early RA Synovium

**Figure 1:**
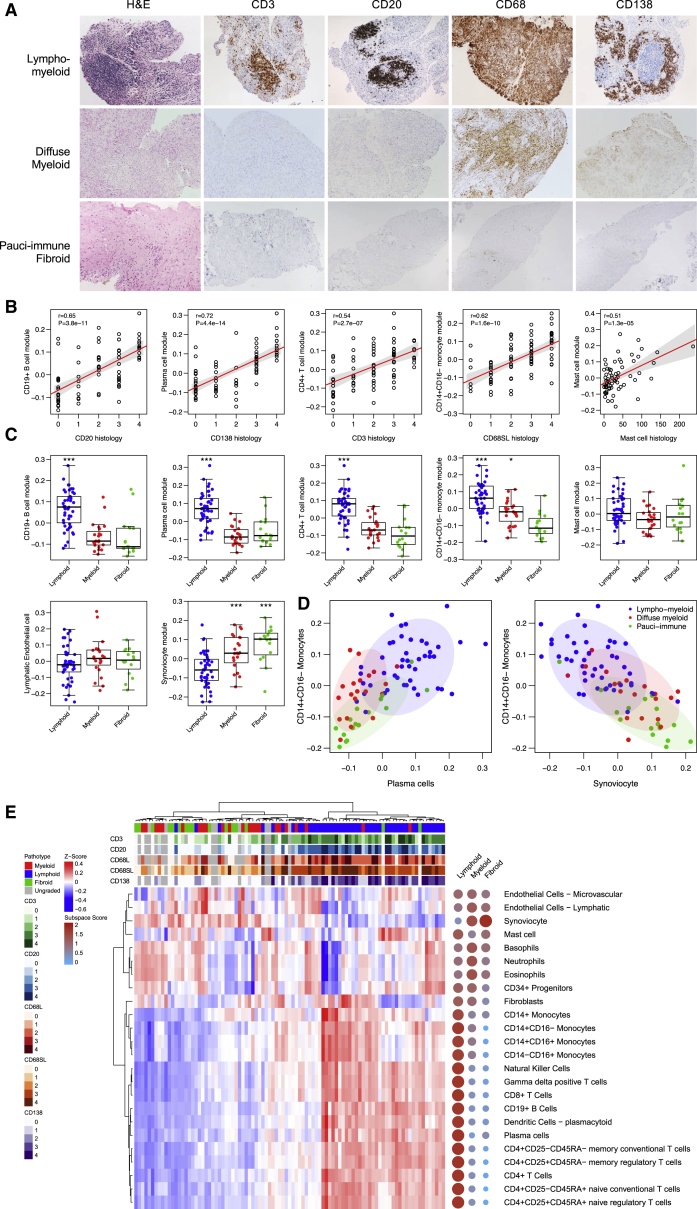
Synovium RNA Sequencing Correlates with Histological Pathotype in Early Rheumatoid Arthritis (A) Immunohistochemistry of synovial biopsies for CD20^+^ B cells, CD3^+^ T cells, and CD68^+^ macrophages in synovial lining or sublining layers and CD138^+^ plasma cells from treatment-naive individuals with early rheumatoid arthritis. Synovial biopsies were categorized as lympho-myeloid (B cell aggregates present), diffuse-myeloid (sublining macrophage infiltration), or pauci-immune fibroid (lack of or low inflammatory cell infiltrate). (B) Comparison of cell-specific RNA-seq gene module scores with histology scores. (C) Cell-specific gene scores compared across histology pathotypes. Statistical analysis by one-way ANOVA with Bonferroni post-test. (D) Clustering of lympho-myeloid, diffuse-myeloid and pauci-immune fibroid samples according to B cell, monocyte, and synoviocyte RNA-seq modules. (E) Heatmap showing hierarchical clustering of cell-specific gene module scores and collapsed module space (right) highlighting cellular composition of synovial biopsies from each pathotype.

Ultrasound-guided synovial biopsies were selected from 90 consecutive individuals (demographics in Table S1) meeting the 1997 ACR classification criteria for early RA from the larger 355-individual PEAC. At presentation, average clinical disease activity was high, with a mean 28-joint disease activity score (DAS28-ESR) of 5.8 ± 1.3 (Table S1). Biopsies were obtained using a minimally invasive ultrasound-guided approach, which we pioneered on a large scale (Kelly et al., 2015) under local anesthesia including both small or medium joints (∼75%) and large joints (∼25%) prior to any therapy with synthetic disease-modifying anti-rheumatic drugs (sDMARDs) including steroids. Synovial biopsies were analyzed by immunohistochemistry and scored semiquantitatively (0–4) for the presence of B cell aggregates (cluster of differentiation [CD]20^+^), plasma cells (CD138^+^), T cells (CD3^+^), and monocytes or macrophages (CD68^+^) in the synovial lining (CD68L) or sublining (CD68SL) layers (Figure 1A). Based on histology scores, synovial samples were classified as lympho-myeloid (CD20 B cell aggregate rich), diffuse-myeloid (CD68 rich in the lining or sublining layer but poor in B cells), or fibroid (paucity of immune-inflammatory cell infiltrate), as described in the STAR Methods. A total of 46 (51%) biopsies were classed as lympho-myeloid, 21 (23%) were diffuse-myeloid, 17 (19%) were pauci-immune fibroid, and 6 (7%) were unclassifiable by histological analysis due to low tissue quality following histology processing (Table S2). Whole-tissue RNA-seq was performed on 90 synovial biopsies, pooling a minimum of 6 biopsies per patient. Three synovial RNA-seq samples were excluded following quality control, resulting in a post-quality control (QC) sample size of 87 synovial RNA-seq samples. RNA-seq performed on matching peripheral blood samples was available on 67 individuals.

Histological pathotype, clinical parameters, and ultrasound analysis were repeated after 6 months, during which individuals were treated with DMARDs, predominantly methotrexate in combination with sulfasalazine and/or hydroxychloroquine (Table S3). Sharp van der Heijde X-ray scores were measured at baseline and after 12 months of treatment.

### Cell-Specific Gene Modules Correlate with Synovial Cellularity by Immunohistology, Confirming the Presence of Pathobiological Endotypes in Early RA Synovitis

We derived gene sets highly specific to immune cell tissue types (Table S4; Figure S1), based on cap analysis gene expression (CAGE) sequencing data from the FANTOM5 project (Forrest et al., 2014), which correlates well with tissue-derived RNA-seq (Yu et al., 2015). RNA-seq gene module scores specific for B cells, T cell subsets, monocyte or macrophage subsets, plasma cells, and mast cells were analyzed for correlation against relevant histological markers in synovial tissue. Gene module scores for CD19^+^ B cells, CD4^+^ T cells, CD14^+^CD16^+^ monocytes, plasma cells, and mast cells correlated strongly with synovial histology scores for CD20 (r = 0.65, p = 3.8 × 10^−11^), CD3 (r = 0.54, p = 2.7 × 10^−7^), sublining CD68 (r = 0.62, p = 1.6 × 10^−10^), CD138 (r = 0.72, p = 4.4 × 10^−14^), and CD117^+^ mast cells (r = 0.51, p = 1.3 × 10^−5^), respectively (Figure 1B). Hence, cell-specific gene modules derived from FANTOM5 data enabled a new method to use RNA-seq data to reveal relative quantitation of tissue immune cell infiltration according to cell-specific transcripts. Cell-specific module scores were compared between the lympho-myeloid, diffuse-myeloid, and pauci-immune fibroid histological groups (Figure 1C); as expected, both the B cell and T cell gene scores were elevated in the lympho-myeloid versus pauci-immune fibroid group (p = 8.7 × 10^−7^ and p = 6.2 × 10^−9^, respectively). The CD14^+^CD16^+^ monocyte gene score was high in both diffuse-myeloid (p = 1.6 × 10^−4^) and lympho-myeloid (p = 2.6 × 10^−9^) groups compared to the pauci-immune fibroid group, whereas the CD138^+^ plasma cell score was highest in the lympho-myeloid group (p = 2.5 × 10^−6^). Thus, plasma cell, B cell, monocyte, and synoviocyte RNA-seq cell-specific modules were able to segregate the histologically defined lympho-myeloid, diffuse-myeloid, and pauci-immune fibroid samples (Figure 1D). In plasma cell-rich synovial samples, we also detected high monocyte or macrophage and T cell module scores (Figure 1D), suggesting a strong association with these cell types most likely to be connected to immunological priming of T cells by APC, leading to B cell activation and differentiation in the synovial tissue. Further analysis of a wider set of immune cell types using gene modules derived from FANTOM5 shows more general patterns of immune cell infiltration in synovial tissue across the 3 major pathotypes (Figure 1E). Gene signatures of CD4^+^ T cell subsets including regulatory T cells, CD8^+^ T cells, plasmacytoid dendritic cells, and natural killer (NK) cells were associated with the lympho-myeloid group, whereas basophil, eosinophil, and neutrophil signatures were more frequently observed in the diffuse-myeloid group. The pauci-immune fibroid group showed increased magnitude of the synoviocyte gene module and, importantly, a distinct absence of immune cells (Figure 1E). Together, the distribution of cell-lineage-specific transcripts (Figures 1D and 1E) suggests that synovial tissue heterogeneity represents a divergent continuum with pauci-immune fibroid samples, low on all types of immune-inflammatory cells at one end of the spectrum and lympho-myeloid at the other end of the spectrum, with the full range of immune-inflammatory cells including macrophage, T, B, and plasma cell infiltration, whereas the diffuse-myeloid samples show a prevalent macrophage infiltration but largely lack T, B, and plasma cell infiltration.

### Cell-Specific Gene Modules in Synovium Correlate with Clinical Phenotypes Featuring Diverse Disease Activity and Radiographic Damage

**Figure 2:**
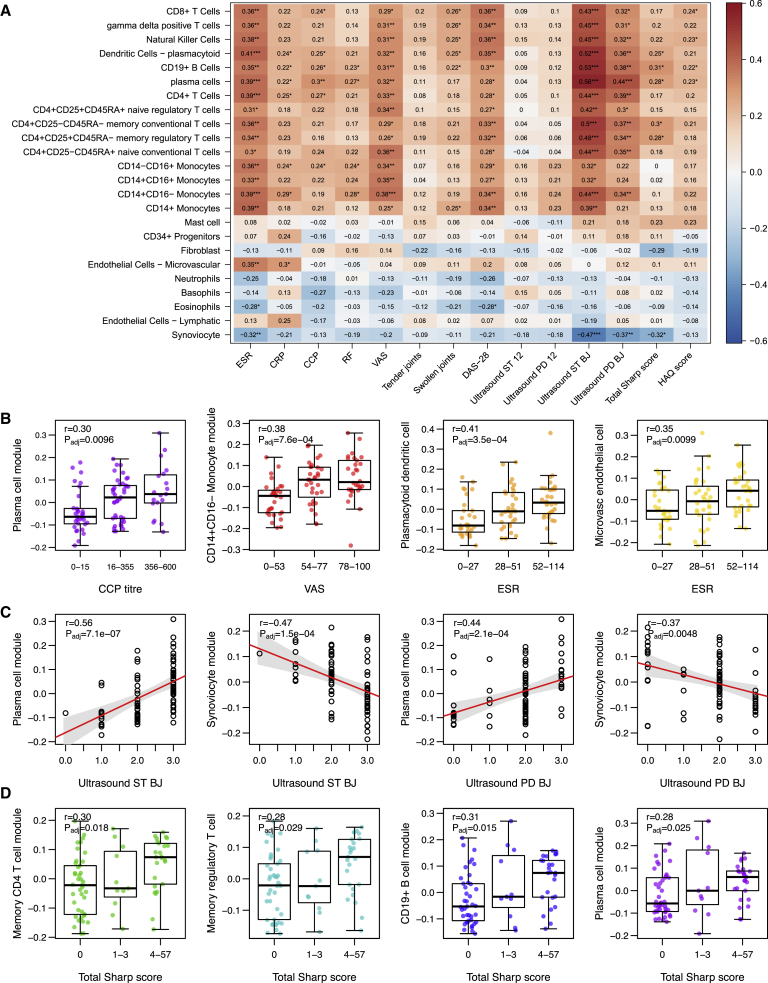
Clinico-radiographic Correlates of Cell-Specific Gene Modules in Rheumatoid Arthritis Synovium (A) Correlation heatmap showing Spearman correlation of cell-specific gene modules against baseline clinical (ESR, erythrocyte sedimentation rate; CRP, C-reactive protein; CCP, anti-cyclic citrullinated peptide antibody titer; RF, rheumatoid factor titer; VAS, visual analog score; HAQ, health assessment questionnaire), ultrasonographic scores (ST, synovial thickness; PD, power doppler) at the biopsy joint (Ultrasound ST/PD BJ) or across 12 representative joints (Ultrasound ST/PD 12) and radiographic parameters (Total Sharp van der Heijde score). (B) Boxplots of clinical parameters by tertile demonstrating correlation with cell-specific gene modules. (C) Linear regression of ultrasound biopsy joint parameters against cell-specific gene modules. (D) Boxplots of total Sharp van der Heijde radiographic score by tertile correlated with cell-specific gene modules. p values were calculated by linear regression models.

Correlation of clinical and radiographic parameters with cell-specific gene modules showed that specific immune cell lineages associate with increased disease activity, which is reflected in the DAS28-ESR including the sedimentation rate (ESR) and tender and swollen joints scores (Figure 2A). The plasma cell gene module showed strongest correlation with anti-cyclic citrullinated peptide (CCP) titer (r = 0.30, false discovery rate [FDR]-adjusted p = 0.0096) (Figure 2B), consistent with previous studies linking synovial B cell infiltration and *in situ* plasma cell differentiation to anti-CCP antibody production (Corsiero et al., 2016, Humby et al., 2009, Teng et al., 2007). CD14^+^CD16^−^ monocyte module correlated with pain visual analog score (VAS) (r = 0.38, p_adj_ = 7.6 × 10^−4^). Plasmacytoid dendritic cell (pDC) (r = 0.41, p_adj_ = 3.5 × 10^−4^) and microvascular endothelial cell modules (r = 0.35, p_adj_ = 0.0099) correlated with ESR, which is consistent with pDC involvement (in addition to myeloid dendritic cell [mDC]) in immune and/or inflammatory responses. Particularly strong correlation was seen between both biopsy joint synovial thickness and power Doppler ultrasonographic measures with gene expression modules, confirming that gene expression of cellular infiltration strongly matches imaging signs of active joint inflammation in the particular joint undergoing biopsy. The plasma cell gene module was the strongest predictor of ultrasonographic synovial thickness (r = 0.56, p_adj_ = 7.1 × 10^−7^) and power Doppler signal (r = 0.44, p_adj_ = 2.1 × 10^−4^) (Figure 2C), which is consistent also with a strong correlation between CD138^+^ histology score and ultrasonography (Figure S2). In contrast, there was an inverse correlation between ultrasound scores and synoviocyte gene expression (pauci-immune fibroid pathotype). Several cell type modules showed significant correlation with radiographic damage, as measured by baseline total Sharp van der Heijde score (Figure 2D): B cell (r = 0.31, p_adj_ = 0.015), CD4^+^ memory T cell (r = 0.30, p_adj_ = 0.018), regulatory T cell (r = 0.28, p_adj_ = 0.029), and plasma cell (r = 0.28, p_adj_ = 0.025) gene signatures were correlated with radiographic change. These data suggest that infiltration of multiple immune cell types associated with ectopic lymphoid responses in the synovial tissue may be linked to more destructive disease from early on in the course of RA.

### Synovium and Blood RNA-Seq Comparison Reveals Differential Axes of Gene Expression

**Figure 3:**
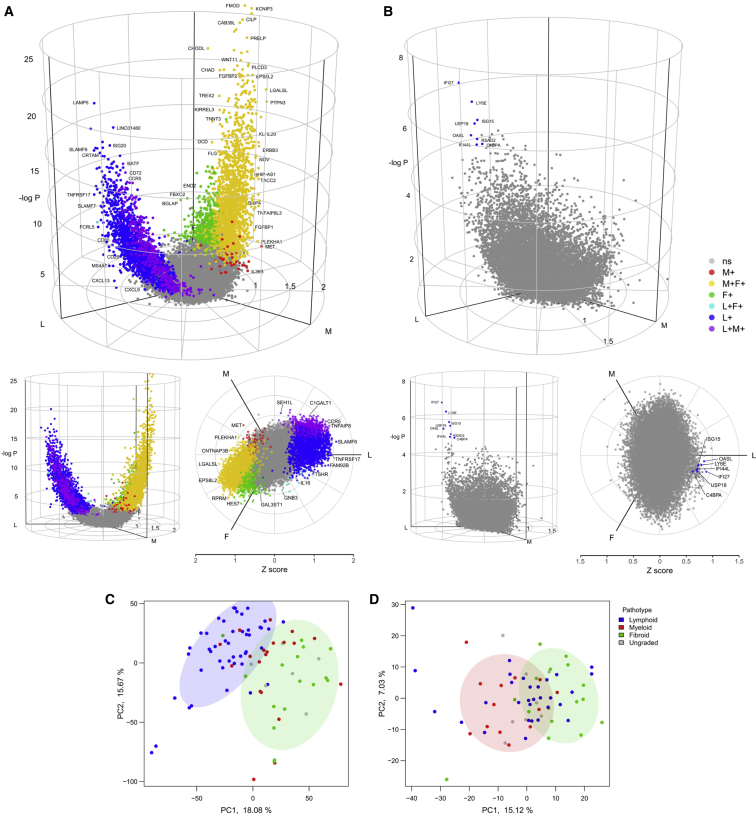
Synovium and Blood RNA-Seq Comparison Reveals Differential Axes of Gene Expression (A and B) 3D cylindrical volcano plots of differentially expressed genes comparing RNA sequencing of (A) synovial tissue and (B) whole blood. Vectors for pathotype mean Z score per gene were projected onto a polar coordinate space analogous to RGB (red-green-blue) color space mapping to HSV (hue-saturation-value) as described in the STAR Methods. Lympho-myeloid, diffuse-myeloid, and pauci-immune fibroid vectors are mapped to 3 axes lympho-myeloid (L), diffuse-myeloid (M), and pauci-immune fibroid (F) using polar coordinates in the horizontal plane. The z axis shows –log_10_ p value for likelihood ratio test. Genes with adjusted p value for likelihood ratio test < 0.05 (z axis) were considered significant (non-significant genes colored gray). Colors demonstrate pairwise comparisons (FDR < 0.05) between the 3 histological pathotypes: primary colors denote upregulation in one group only for lympho-myeloid (blue), diffuse-myeloid (red), and pauci-immune fibroid (green) compared to reference group with minimum gene expression; composite colors show genes significantly upregulated in two groups (myeloid+lymphoid, purple; fibroid+myeloid, yellow; lymphoid+fibroid, cyan). Lateral view and 2D polar plots are shown below. (C and D) Principal-component analysis of whole transcriptome RNA-seq data from untreated rheumatoid arthritis (C) synovium and (D) whole blood, showing separation of lympho-myeloid (blue) and pauci-immune fibroid (green) histological pathotypes on principal component 1 (PC1) for synovial RNA-seq, with separation of diffuse-myeloid (red) and pauci-immune fibroid samples on PC1 in whole blood.

We next compared gene expression in synovium and peripheral blood in the three histologically identified subgroups using FDR-adjusted likelihood ratio test and pairwise group tests for differential expression. Differentially expressed genes were initially visualized using standard volcano plots (Figure S3). However, due to the three-way nature of the analysis, the multiple pairwise comparisons rendered data interpretation difficult. Hence, we developed a 3D volcano plot by using a cylindrical geometry to aid visualization and interpretation of the three-way group comparison (Figures 3A and 3B; Videos S1 and S2). The three-way volcano plots demonstrate that the largest groups of differentially expressed RA synovium genes are upregulated in the lympho-myeloid group alone (blue) or diffuse-myeloid and pauci-immune fibroid combined (yellow), with a smaller number of genes associated with diffuse-myeloid group alone (red) (Figure 3A). The polar angle of each gene directly conveys the degree to which a gene is associated with one or more pathotypes. Fold change can be used as an alternative to Z score for the radial scale (see online https://peac.hpc.qmul.ac.uk).

Video S1. Three-Way Volcano Plot of Synovial RNA-Seq Genes Differentially Expressed between Histological Pathotypes, Related to Figure 3A

Video S2. Three-Way Volcano Plot of Blood RNA-Seq Genes Differentially Expressed between Histological Pathotypes, Related to Figure 3B

Comparison of synovium and blood RNA-seq showed a stark difference in the absolute quantity of differentially expressed transcripts between pathotypes, with approximately 3,000 transcripts in synovium compared to only 8 differentially expressed transcripts in matched peripheral blood at FDR < 0.05 (Figure 3B). The eight differentially expressed blood transcripts were associated with the lympho-myeloid pathotype, and seven out of eight are known type I interferon response genes (*IFI27*, *ISG15*, *IFI44L*, *OASL*, *USP18*, *RSAD2*, and *LY6E*). The overall transcriptome shape as visualized in the polar plots (Figures 3A and 3B) showed greater whole-transcriptome variation between the lymphoid-fibroid axis in synovium in comparison to the myeloid-fibroid axis in blood. Pairwise volcano plots (Figure S3) confirmed the largest number of differentially expressed genes in peripheral blood were seen in the myeloid-fibroid comparison, but, in contrast, in synovium the myeloid-fibroid comparison showed the fewest number of differentially expressed genes.

To facilitate the interrogation of this large synovial tissue RNA-seq database, we developed a web interface (https://peac.hpc.qmul.ac.uk/) that facilitates visualization and exploration of the data (Figure S4). The web interface includes an interactive version of the 3D volcano plot; an interactive interface for comparing genes or gene modules of interest in synovium or blood against histological, clinical, or radiographic measures; and searchable tables of differentially expressed genes and module comparisons.

To find evidence of natural structure in the transcriptome data, principal-component analysis (PCA) of synovium was compared with whole blood (Figures 3C and 3D). Synovial transcriptome PCA showed clear separation between lympho-myeloid and pauci-immune fibroid groups, whereas the diffuse-myeloid partially overlapped with the two groups. However, in the whole-blood transcriptome, PCA showed separation between patients showing a diffuse-myeloid and pauci-immune fibroid synovial pathotype on PC1, whereas patients with the lympho-myeloid synovial pathotype were evenly distributed. Taken together, the clustering analysis and PCA suggest that although the synovium gives clean delineation of the lympho-myeloid group, particularly in those individuals with synovial plasma cells, the blood transcriptome shows significantly less differentiation between pathotypes.

### Synovial RNA-Seq Gene Clusters Delineate Pathways Characterizing Histo-pathotype Spectrum

**Figure 4:**
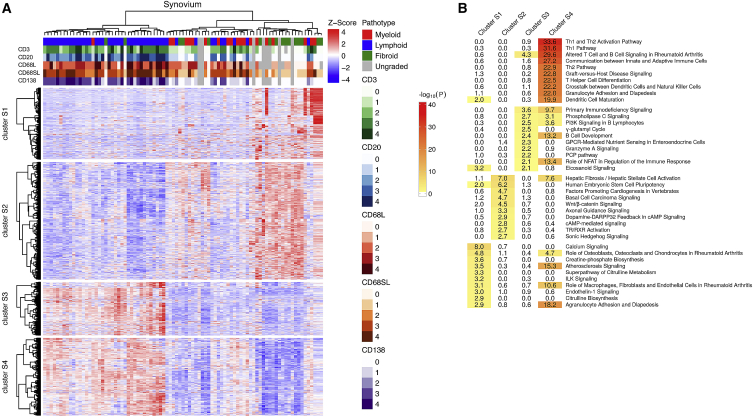
Clustering and Pathway Analysis of Differentially Expressed Genes in Rheumatoid Arthritis Synovium (A) Heatmap of 2,964 RNA-seq genes differentially expressed between three histological pathotypes (lympho-myeloid, diffuse-myeloid, and pauci-immune fibroid) (FDR < 0.05, n = 87). Upper tracks show histological scores for CD3, CD20, CD68L, CD68SL, and CD138 and overall pathotype. Unsupervised hierarchical clustering demonstrated clustering of genes into four clusters, demonstrating some overlap between the three histologically determined pathotypes. (B) Ingenuity Pathway Analysis performed on synovial gene clusters produced by hierarchical clustering identified pathways by gene enrichment, using whole genome as background. Clusters S1 and S2 represent pauci-immune fibroid and diffuse-myeloid samples, and clusters S3 and S4 represent lympho-myeloid and diffuse-myeloid samples. Color scale and numbers depict –log_10_ FDR-adjusted p values.

Differentially expressed genes in RA synovial biopsies were subjected to unsupervised hierarchical clustering and compared against histology (Figure 4A). Cluster S1 and S2 were mainly associated with pauci-immune and diffuse-myeloid samples, whereas S3 and S4 were typically associated with more inflamed diffuse-myeloid or lympho-myeloid samples. In comparison, little evidence of structure and relationship to pathotypes was observed in clustering of the top ∼500 variable genes in blood (data not shown). Biological processes for each synovial and blood gene cluster were investigated using ingenuity pathway analysis (IPA) (Figure 4B). Overall, pathway analysis showed strong segregation, concordant with histology. The strongest pathway enrichment was identified in cluster S4 (lympho-myeloid group), which was associated with both B cell, plasma cell, and macrophage infiltration histologically, and demonstrated multiple immune cell activation, encompassing B and T helper cell maturation associated with dendritic cell activation, antigen presentation, and interaction with NK cells typically associated with ectopic lymphoid-like structure (ELS) formation. Cluster S3 pathways showed specific pro-inflammatory pathways including phospholipase C, PI3K, and NFAT signaling, which are known to be important drivers of activation and infiltration of tissue neutrophils, macrophages, and other immune cell types into inflamed synovium (Jakus et al., 2009). In contrast, clusters S1 and S2, which were dominated by pauci-immune and diffuse myeloid samples, were associated with pro-fibroblast Wnt/β-catenin pathways, whereas immune and/or inflammatory pathways were notably lower.

### Modular Analysis Shows Discordance between Blood and Synovium Immunological Pathways

**Figure 5:**
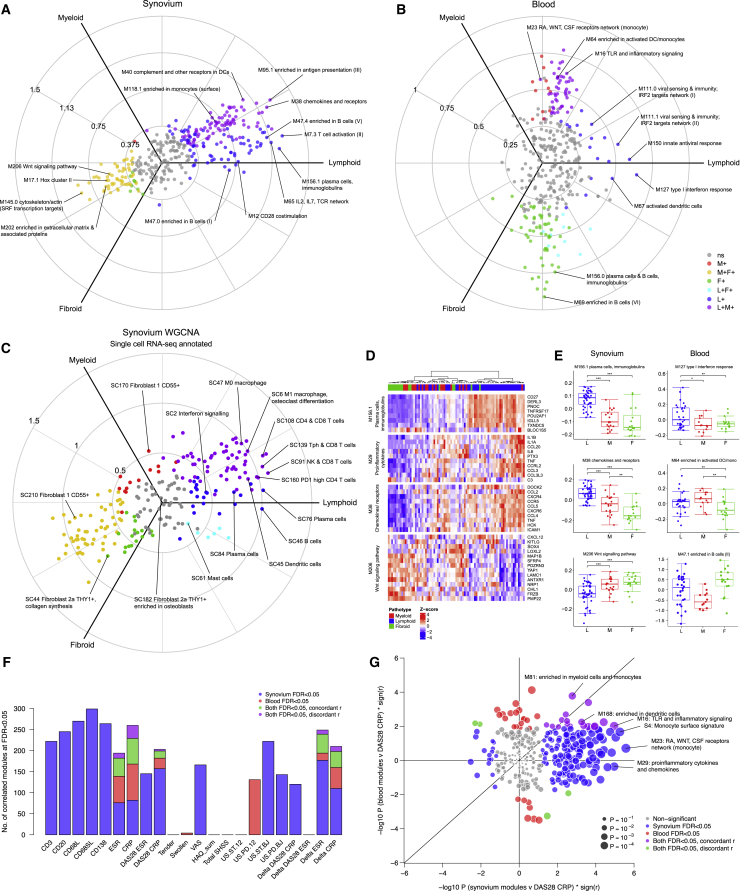
Modular Analysis of Synovial and Blood RNA Sequencing (A–C) Three axis polar plots of synovium (A) and blood (B) gene modules based on blood microarray modules (Li et al., 2014) and (C) synovium modules derived by weighted correlation network analysis (WGCNA), analyzed using QuSAGE. Modules are color-coded for statistical significance (FDR < 0.05) for upregulation in different pathotypes. WGCNA synovium modules were annotated against single-cell RNA-seq cell types (Stephenson et al., 2018). (D) Heatmap showing gene expression in selected gene modules in synovium and blood, grouped by pathotype. (E) Boxplots of summarized module scores in synovium and blood. Statistical analysis by QuSAGE with FDR correction: ^∗^FDR < 0.05, ^∗∗^FDR < 0.01, ^∗∗∗^FDR < 0.001. (F) Comparison of number of significant (FDR < 0.05) synovium and blood gene modules (Li et al., 2014), which correlate with clinical and radiographic markers of disease activity and response to 6 months treatment with DMARDs, in either synovium only (blue) or blood only (red) or are concordantly correlated in both compartments (purple). Statistical analysis by Spearman correlation. (G) Bubble plot of –log_10_ p values comparing correlation of gene modules in synovium and blood with disease activity measured by DAS28-CRP, showing significantly (FDR < 0.05) correlated modules found in synovium alone (blue), blood alone (red), or concordantly correlated with DAS28-CRP in both synovium and blood (purple).

Blood transcript modules (Li et al., 2014) were applied to the RA synovium and peripheral blood transcriptome data. We used Quantitative Set Analysis for Gene Expression (QuSAGE) methodology to compare differential gene module expression between synovial pathotypes in synovium (Figures 5A and S5A) and peripheral blood (Figures 5B and S5B). In synovium, plasmal, B, and T cell gene modules were strongly upregulated in the lympho-myeloid pathotype, as were gene modules for CD28 costimulation, interleukin-7 (IL-7) and B and T cell differentiation (Figures 5A and S5A). Pro-inflammatory chemokine and cytokine modules and dendritic cell modules were associated with both diffuse-myeloid and lympho-myeloid pathotypes, in keeping with their role in monocyte recruitment and macrophage activation in the diffuse-myeloid pathotype and ectopic lymphoid structure development in the lympho-myeloid pathotype. Pauci-immune fibroid synovial modules were enriched for cell junction, cell-cell adhesion, extracellular matrix, and Hox cluster, which is consistent with fibroblast and connective tissue development.

To complement this analysis, we performed weighted correlation network analysis (WGCNA) on synovium RNA-seq. Synovium WGCNA modules (Figure 5C; Table S5) were annotated against 13 cell populations observed in single cell RNA-seq of RA synovium (Stephenson et al., 2018), by testing for module gene enrichment in genes upregulated in each cell type by hypergeometric test. Correlation of synovium WGCNA modules with clinical variables showed comparable results to FANTOM5 cell-lineage modules (Figure S6; and see https://peac.hpc.qmul.ac.uk/) and provides additional information on synovial cell types not available in FANTOM5, including synovial fibroblast subtypes (Mizoguchi et al., 2018) and synovial macrophage and T cell subpopulations including peripheral helper T cells (T_PH_ cells) (Rao et al., 2017). The single-cell plasma cell module showed similar results to FANTOM5 (Figure S6A). The SC160 module, which contains multiple genes associated with PD-1^high^ T_PH_ cells including *PDCD1*, *TIGIT*, and *CXCR6*, showed strong correlation with increased disease severity measured by DAS28-ESR, ultrasound at the biopsy joint, and Sharp van der Heijde radiographic damage score (Figure S6B). A CD55^+^ type 1 fibroblast-associated module, SC210, was strongly associated with the pauci-immune fibroid pathotype and was inversely correlated with inflammatory cell histology and showed inverse correlation with disease severity measured by multiple clinic-radiographic parameters (Figure S6C).

Heatmaps of individual gene modules revealed that plasma-cell-associated genes such as *CD27* and *IGLL5* were strongly upregulated in lympho-myeloid samples, whereas multiple chemokine genes were increased in both lympho-myeloid and diffuse-myeloid samples (Figures 5D and 5E). In contrast, Wnt signaling module M206 (Figures 5D and 5E), containing *FRZB*, which has a critical role in bone and cartilage development, was elevated in the pauci-immune fibroid pathotype (Figures 5D and 5E).

The polar plot of the blood module signatures (Figure 5B) was distinctively different from synovium, with the main axis of variation lying from myeloid to fibroid, mirroring the distinct whole-transcriptome variation between the two compartments (Figures 3A and 3B, polar plots). Noteworthy, blood module associations with pathotype included type I interferon response and paradoxical changes in the B cell compartment. Increased peripheral blood type I interferon response (M127), which is involved in terminal B cell maturation and plasma cell development, was associated with the lympho-myeloid pathotype in synovium (p_adj_ = 0.002) (Figures 5B, 5E, and S5B), which is in line with our earlier differential expression data (Figure 3B). Blood B cell modules were reduced in the diffuse-myeloid pathotype, which could represent B cell flux into tissues leading to reduced circulating peripheral B cells (Figure 5E).

We directly compared differences between synovium and blood, looking at the number of gene modules in each compartment that significantly correlated with histology, clinical, and radiological variables at FDR < 0.05 (Figure 5F). Synovial modules were strongly correlated with histology, acute phase response, VAS, and overall disease activity measured by DAS28-ESR/C-reactive protein (CRP). Synovium modules also strongly correlated with ultrasound power Doppler and synovial thickness at the biopsy joint. Blood module associations with clinical parameters were generally rarer than for synovium, except for strong correlation with blood markers of acute phase response such as ESR and CRP. Looking specifically at disease activity measured by DAS28-CRP (Figure 5G), substantially more synovium modules correlated with DAS28-CRP than for blood; however, a few monocyte and dendritic cell modules such as M81, M168, and toll-like receptor (TLR) module M16 showed correlation with DAS28-CRP in both synovium and blood.

### Synovium Plasma Cell Gene Expression Is Associated with Anti-CCP Antibody Positivity and Predicts Worse Prognosis at 12 Months

**Figure 6:**
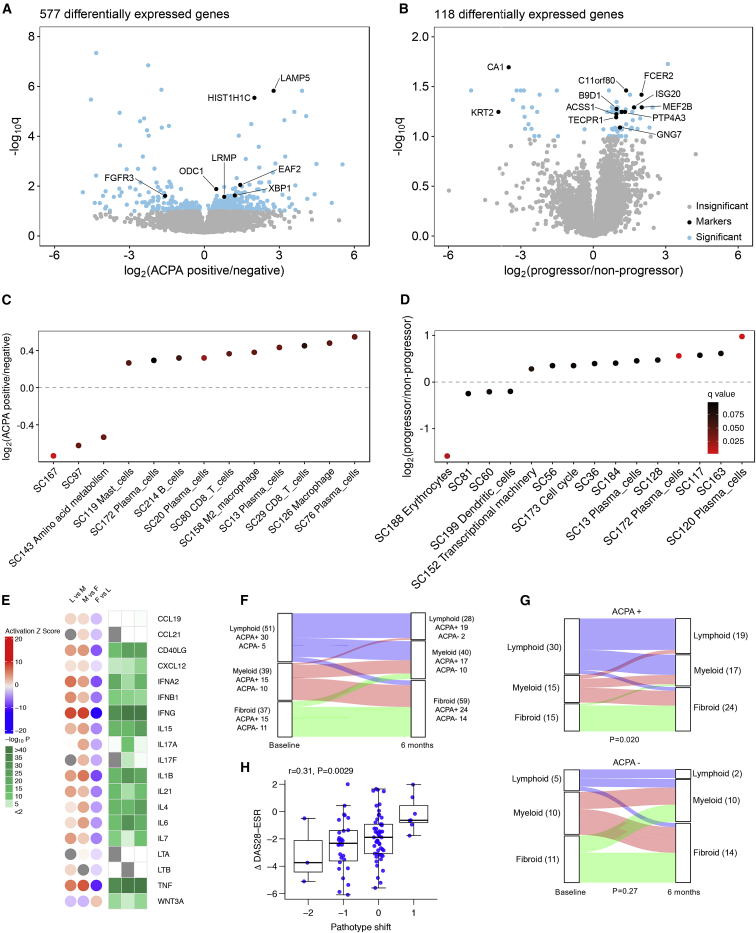
Baseline Synovium Plasma Cell Gene Expression Is Associated with CCP Antibody Positivity and Worse Prognosis at 12 Months (A and B) Differential gene expression in synovium RNA-seq comparing (A) anti-CCP antibody (ACPA)-positive and ACPA-negative RA individuals and (B) individuals with progression of bone erosions on X-rays at 12 months compared to baseline versus non-progressors. (C and D) Single-cell RNA-seq-annotated WGCNA modular analysis shows that increased plasma cell module expression is associated with ACPA positivity (C) and is predictive of bone erosion radiographic progression at 12 months (D). (E) Upstream regulator analysis using Ingenuity Pathway Analysis showing upstream regulator effects of cytokines and chemokines associated with ectopic lymphoid structure development in synovium. (F and G) Sankey diagrams showing change in histological pathotype following 6-month treatment with disease-modifying anti-rheumatic drugs (DMARDs) for (F) whole cohort or (G) grouped by ACPA status. Statistical analysis by Fisher’s test. (H) Shift in pathotype between baseline and 6 months correlated against change in DAS28-ESR. Statistical analysis by Pearson correlation.

Differential gene expression between anti-CCP antibody (ACPA)-positive and ACPA-negative individuals showed increased plasma cell genes such as *XBP1*, *ODC1*, and *EAF2* as well as *LAMP5*, a regulator of TLR9 in pDCs (Combes et al., 2017), which was the strongest pro-lympho-myeloid pathotype gene (Figure 6A). In contrast, although relatively few genes were differentially expressed in blood between ACPA positive versus negative individuals, these included type I interferon response genes *IFI44L* and *IFI27* (Figure S7), which is consistent with a blood type I interferon signature underlying synovial plasma cell infiltration. RA individuals with X-rays at baseline and 12-month follow-up were divided into progressors, in whom radiographic bone erosions had worsened over 12 months, or non-progressors. Plasma-cell-associated genes were significantly increased in bone erosion progressors compared to non-progressors, demonstrating that synovial plasma cell gene expression at baseline predicts a worse prognosis radiographically at 12 months. Single-cell RNA-seq-annotated WGCNA modular analysis showed that ACPA positivity was associated with increased plasma cell and macrophage gene modules (Figure 6C), and baseline plasma cell modules also predicted bone erosion progression at 12 months (Figure 6D).

### Transcriptional Regulators of Ectopic Lymphoid Structure Development

Upstream regulator analysis using IPA (Figure 6E) showed that key regulators including interferon gamma (IFN-γ), IFN-α2, IFN-β1, IL-7, IL-21, and CD40L were associated with the lympho-myeloid pathotype, which is consistent with the dominant theme of B cell proliferation, differentiation, and plasma cell development (Hiepe et al., 2011), and the previously reported association of IL-7 pathway with synovial B cell infiltration (Badot et al., 2009, Timmer et al., 2007). The follicular helper T cell cytokine IL-21 is important for ELS maturation (Jones et al., 2015, Jüngel et al., 2004, Liu et al., 2015). We also confirmed the association of the chemokine CXCL12 with ELS formation (Timmer et al., 2007), which is consistent with its role in maintaining long-lived plasma cells (Tokoyoda et al., 2004). TNF, IFN-γ, IL-1β, IL-4, IL-6 ,and IL-15 were upstream regulators for the diffuse myeloid pathotype, whereas WNT3A was an upstream regulator of the pauci-immune fibroid pathotype.

Histological pathotype was re-analyzed after 6 months (Figure 6F), during which individuals were treated with DMARDs (Table S3). In ACPA-positive RA individuals, the histological pathotype showed a more consistent tendency (p = 0.020) to change to a less inflammatory pathotype, i.e., from lymphoid to myeloid, or myeloid to fibroid (Figure 6G), whereas in ACPA-negative individuals change in pathotype at 6 months was less consistent (p = 0.27). Stratifying DAS28-ESR measurements according to whether individuals had shifted to a more or less inflammatory pathotype from baseline to 6 months, we observed a linear relationship between shift in pathotype and change in DAS28-ESR (r = 0.31, p = 0.0027) (Figure 6H). Thus, individuals, whose pathotype shifted to a less inflammatory pathotype at 6 months, e.g., from lymphoid to myeloid or lymphoid to fibroid, showed significantly greater reductions in DAS28-ESR, whereas individuals whose pathotype became more inflammatory, e.g., from fibroid to myeloid, on average showed no reduction in disease activity. Thus, changes in synovial pathotype, as detected by serial synovial biopsy, reflect clinical responsiveness to DMARD therapy. This is consistent with our assertion that the complex autoimmune milieu observed within RA synovial tissue directly underlies core pathogenic processes that drive RA progression, and when improved therapeutically, the change in this autoimmune milieu is detectable in sequential biopsies.

### Baseline Predictors of Response to Treatment

**Figure 7:**
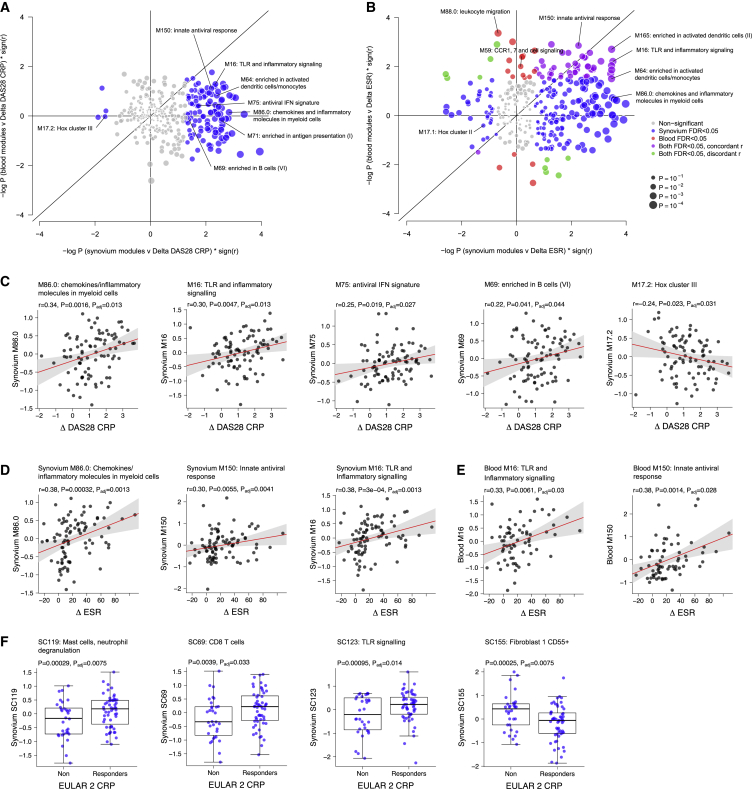
Association of RNA-Seq Modules and Response to 6 Months of DMARD Treatment (A and B) Correlation of gene modules in synovium (x axis) versus blood (y axis) with 6-month response to DMARD treatment measured by (A) delta DAS28-CRP and (B) delta ESR. Significantly correlated synovial modules (at FDR < 0.05) are shown in blue, significant blood modules in red, and modules, which concordantly correlate with each clinical parameter in both synovium and blood, are shown in purple. (C) Correlation of synovium gene modules with delta DAS28-CRP from baseline to 6 months. (D and E) Correlation of (D) synovium and (E) blood gene modules against change in ESR from baseline to 6 months following DMARD treatment. Statistical analysis by Spearman correlation with FDR adjustment (A–E). (F) Differential expression of synovial single-cell-annotated WGCNA modules between EULAR DAS28-CRP responders (good and moderate) and non-responders. Statistical analysis by QuSAGE with FDR adjustment.

Numerous synovial modules correlated with response to treatment after 6 months measured by change in DAS28-CRP, including type I IFN signature and antiviral modules, monocyte and chemokine modules, dendritic cell and antigen presentation modules, and B cell modules (Figures 7A and 7C), demonstrating that a more inflammatory synovial phenotype at baseline correlated with a greater fall in DAS28-CRP after 6 months of DMARD treatment. However, no blood modules were associated with clinical response. Looking at the acute phase response (Figure 7B), we observed that both synovium and blood modules were associated with reduction in ESR, with some modules such as TLR signaling, antiviral response, and dendritic cell modules showing association with ΔESR in both compartments (Figures 7B, 7D, and 7E). In contrast, pauci-immune-fibroid-associated Hox cluster modules were associated with resistance to treatment. These findings suggest that although blood gene expression directly reflects the systemic inflammatory and acute phase response and, thus, is associated with change in ESR or CRP following treatment, blood gene expression is not a strong predictor of overall clinical response. Grouping patients by European League Against Rheumatism (EULAR) response using DAS28-CRP, we observed that modules for CD8^+^ T cells, mast cells, and TLR signaling were significantly increased in EULAR moderate and good responders at 6 months compared to non-responders, whereas a CD55^+^ type 1 fibroblast module was lower in responders (Figure 7F), although pathotype per se was not significantly different between EULAR response groups. Thus, specific synovial cell types are associated with differential response to DMARD treatment.

## Discussion

This study represents the most comprehensive exploration of synovial and blood RNA-seq in RA to date, which combines detailed histopathological classification and in-depth clinical phenotyping. The interactive website (https://peac.hpc.qmul.ac.uk/) developed with these data will allow the research community to dissect synovial pathology architecture, allowing researchers to explore the data and correlate genes and gene modules with histological, clinical, and radiographic parameters. Based on our previous work using histopathology, we identified three partially overlapping groups: (1) a lympho-myeloid (L) group showing aggregates of B and T lymphocytes associated with diverse inflammatory cell infiltrate; (2) a diffuse-myeloid (M) group characterized by macrophage or monocyte enrichment, but poor in B cells; and (3) a pauci-immune fibroid (F) group showing a distinct lack of immune-inflammatory infiltrate. Multiple techniques were used to investigate links between synovium and blood transcriptomes to further define the pathobiological subtypes.

We developed and validated cell-lineage specific gene modules derived from FANTOM5 data for relative quantitation of cell populations in RNA-seq tissue samples. This transcript-based immune cell phenotyping performed well in comparison to established immunohistology (Humby et al., 2009). The analysis suggested that the immune cell infiltration in synovium is a continuum with pauci-immune fibroid pathotype, lacking immune cell infiltration at one end, and the lympho-myeloid pathotype with diverse immune cell infiltration with NK cells and plasmacytoid DC, with fully formed ELS and high levels of plasma cells at the most advanced end of the spectrum. Macrophage infiltration appeared to be a prerequisite for B cell activation and plasma cell development, implying their potential for local differentiation into antigen-presenting cells (APC) driving T cell activation, T follicular helper cell (Tfh) activation, and formation of ELS. Although our data support the notion that the pathotypes represent different types of synovitis, with differing pathogenic process and inflammatory milieu, we cannot exclude the possibility that they represent evolving states of activation or gear shifts in the disease process.

Correlation of cell-specific RNA-seq gene modules with clinical and radiological parameters showed that the multiple synovial immune cell types including monocytes or macrophages were correlated with disease activity measured by DAS28-ESR. The plasma cell gene module correlated with ACPA and rheumatoid factor titer, which is consistent with the notion that local *in situ* plasma cell differentiation and perpetuation are associated with high ACPA titer in RA (Corsiero et al., 2016, Teng et al., 2007). Multiple cell modules were correlated with ultrasonographic scores at the biopsy joint, with particularly strong correlation for the plasma cell transcript module but inverse correlation with the synoviocyte module. Similarly, radiographic joint damage at baseline correlated with CD4^+^ T, T_reg_, B, and plasma cells. Historically, previous studies identified an association between synovial macrophage infiltration and radiographic progression (Bresnihan et al., 2009, Yanni et al., 1994). In our study, gene transcript changes in synovium largely reflect the altered cellularity in synovium in line with pathogenic processes in RA. The data suggest that disease severity, as measured by ultrasonographic and radiographic change, correlates with advanced synovial immune cell infiltration and *in situ* plasma cell development.

Recent RNA-seq studies of synovium in established, long-standing RA have identified novel cell populations including PD-1 high T_PH_ cells (Rao et al., 2017) and distinct fibroblast subsets (Mizoguchi et al., 2018). As a counterpart to using FANTOM5, we leveraged single-cell RNA-seq data to annotate WGCNA synovial gene modules according to 13 cell populations identified in single-cell RNA-seq of RA synovium (Stephenson et al., 2018). The single-cell RNA-seq-annotated gene modules showed comparable results to the FANTOM5 approach and add additional information about other important synovial cell types not available in FANTOM5, including fibroblast, macrophage, and T cell subpopulations (Figure 5C). Although single-cell RNA-seq of synovial biopsies would have allowed more complete gene expression deconvolution at the level of individual cells, this approach would have been difficult to apply to large numbers of samples, and the reduced dynamic range of single-cell RNA-seq impairs quantitation of rare, low-abundance transcripts. In the future, ongoing efforts in collaboration with the NIH-funded Accelerating Medicines Partnership (AMP) combining both methodologies may yield further insights.

Synovial RNA-seq data demonstrated distinct gene clustering with significantly different gene sets upregulated between the three pathological groups. We developed an interactive 3D volcano plot, as part of our website resource (https://peac.hpc.qmul.ac.uk/), to illustrate the three-way differences in synovial gene expression. Differential gene expression in blood was far less extensive than in synovium but revealed type I IFN response genes associated with the presence of the lympho-myeloid pathotype in synovium. Similar type I IFN response genes have been associated with differential response to Rituximab (Raterman et al., 2012, Vosslamber et al., 2011). Synovium and blood compartments demonstrated differential axes of gene expression variation. Synovium gene expression showed strongest delineation along the lymphoid-fibroid axis, whereas the blood whole transcriptome showed a tendency to delineate pauci-immune fibroid from diffuse-myeloid development. A potential implication is that the adaptive immune response is localized to the site of inflammation or secondary lymphoid organs. Secondary amplification of the myeloid system by cytokines such as type I IFN may lead to a signature in the blood. Cluster analysis of pathotype-specific genes identified pro-inflammatory genes common to both the lympho-myeloid and diffuse-myeloid groups with pathways consistent with infiltration and differentiation of multiple cell types, including T helper cells and dendritic cells, whereas genes that were more specific to B cell differentiation, including PI3K signaling were associated with B cell and plasma cell infiltration histologically.

We did not perform simultaneous biopsies of different joints in the same patient, so we cannot address the question of how stable synovial histology and RNA expression are between joints within the same patient. However, other studies support the notion of stable cellular infiltrates (Kraan et al., 2002) and T cell clonality (Musters et al., 2018) between joints. Synovial histology and RNA expression directly reflect the inflammatory state within the biopsied joint, so our finding that specific immunological processes in peripheral blood, such as the type I IFN response, were associated with synovial B cell infiltration supports the notion of the RA disease process driving a consistent immunological response in each tissue compartment.

Baseline synovial plasma cell gene modules were associated with ACPA positivity and worse prognosis in terms of radiographic damage at 12 months (Figure 6). Local *in situ* plasma cell differentiation and persistence of long-lived tissue plasma cells are likely to play an important role in ACPA formation and perpetuation of disease. Upstream regulator analysis (Figure 6E) identified multiple regulators known to facilitate ectopic lymphoid structure development within tissues, which is consistent with local B cell maturation and plasma cell differentiation being key events underlying the lympho-myeloid pathotype.

Following treatment with DMARDs, the shift in synovial pathotype on repeat biopsy at 6 months correlated with clinical response to DMARD therapy (Figures 6F–6H). Individuals whose pathotype became less inflammatory (e.g., lymphoid to myeloid or lymphoid to fibroid) showed greater reduction in disease activity, whereas individuals whose pathotype progressed to a more inflammatory state showed little or no response. We observed that high baseline inflammatory synovial gene modules including TLR signaling, type I IFN signature, and macrophage chemokine modules were associated with better response at 6 months to DMARDs, as assessed by ΔDAS28-CRP, whereas blood modules were only associated with reduction in ESR at 6 months. Thus, baseline blood gene expression can, to a limited extent, anticipate changes in the systemic acute phase response in response to DMARD therapy but are less informative than synovium gene expression for predicting clinical outcome, which includes swollen and tender joint count and VAS.

Although disease heterogeneity has long been postulated in RA, our study provides the clearest map to date of the relationship between peripheral blood signals and development of different patterns in the synovium early in the RA disease process prior to therapeutic intervention, while avoiding the confounding effects of therapy, especially corticosteroids, on disease tissue pathology. Our data advance our understanding of RA pathogenesis, revealing major differences in synovial gene expression across the histo-pathotype spectrum, and identifying associated pathways and gene modules for each pathotype. Although there was substantially less variation in the peripheral blood transcriptome than in synovium, a few identifiable blood transcript signals were linked to clinical measures of disease activity (monocyte activation and TLR signaling). While synovium, both histologically and at the gene expression level, was highly informative for its association with disease activity and disease progression, blood gene expression independently revealed systemically altered gene expression in the form of upregulated type I IFN signature linked to pathogenic plasma cell infiltration into synovium. Overall, synovial modules were superior for predicting clinical response to DMARD therapy at 6 months and poor prognosis in terms of radiographic progression at 12 months.

In summary, we report an in-depth RNA-seq analysis of synovial tissue and peripheral blood in early RA, prior to therapeutic modification of the disease pathology, and linked to detailed phenotypic profiling. Our data suggest that persistent synovial plasma cell infiltration identifies individuals at increased risk of rapid disease progression and severe joint destruction, and provide the strongest evidence yet that optimal stratification of RA therapies would be enhanced by sampling of both synovium and blood biomarkers.

## STAR★Methods

### Key Resources Table

**Table:** 

REAGENT or RESOURCE	SOURCE	IDENTIFIER
**Antibodies**		

CD20cy, L26, Unconjugated, Culture supernatant	Agilent Technologies	Dako Cat# M0755 RRID:AB_2282030
CD3, F7.2.38, Unconjugated, Culture supernatant	Agilent Technologies	Agilent Technologies Cat# M7254 RRID:AB_2631163
CD68, KP1, Unconjugated, Culture supernatant	Agilent Technologies	Dako Cat# M0814 RRID:AB_2314148
CD138, MI15, Unconjugated, Culture supernatant	Agilent Technologies	Dako Cat# M7228 RRID:AB_2254116
CD21, 1F8, Unconjugated, Culture supernatant	Agilent Technologies	Dako Cat# M0784 RRID:AB_2085307

**Chemicals, Peptides, and Recombinant Proteins**		

TRIzol® Reagent	ThermoFisher Scientific/Invitrogen Division	15596018
Xylene Mixt. of Isomers ANALAR ACS/R.PE - Analytical Grade	VWR International	28975.325
Eosin Yellowish	VWR International	341973R
Haemotoxylin	Sigma-Aldrich	H-3136

**Deposited Data**		

FANTOM5 CAGE seq data	Forrest et al., 2014	http://fantom.gsc.riken.jp/
RNA-seq on synovium and blood in rheumatoid arthritis	This paper	ArrayExpress E-MTAB-6141

**Software and Algorithms**		

R statistics	R foundation	https://www.r-project.org/
Kallisto	Bray et al., 2016	https://pachterlab.github.io/kallisto/
tximport	Bioconductor	https://www.bioconductor.org/packages/release/bioc/html/tximport.html
DESeq2	Love et al., 2014	https://www.bioconductor.org/packages/release/bioc/html/DESeq2.html
shiny	RStudio	https://shiny.rstudio.com/
plotly.r	Plotly	https://plot.ly/r/
ComplexHeatmap	Bioconductor	https://www.bioconductor.org/packages/release/bioc/html/ComplexHeatmap.html
Ingenuity Pathway Analysis	QIAGEN Bioinformatics	https://www.qiagenbioinformatics.com/IPA
QuSAGE	Yaari et al., 2013	https://www.bioconductor.org/packages/release/bioc/html/qusage.html
WGCNA	Langfelder and Horvath, 2007	https://cran.r-project.org/web/packages/WGCNA/index.html
Cytoscape	Cytoscape Consortium	https://www.cytoscape.org/
PEAC RNA-seq web interface	This paper	https://peac.hpc.qmul.ac.uk/

**Other**		

Target Retrieval Solution, x10 Concentrate	Agilent Technologies	S1699
DAB+, Liquid, 2-component system	Agilent Technologies	K3468
Peroxidase-Blocking Solution, Dako REAL	Agilent Technologies	S2023
Protein Block, Serum-Free, Liquid form,	Agilent Technologies	X0909
Antibody Diluent, Background Reducing	Agilent Technologies	S3022
EnVision+ Single Reagents, HRP. Mouse	Agilent Technologies	K4001
Proteinase K	Agilent Technologies	S3020
RNA 6000 Nano Kit	Agilent Technologies	5067-1511
Microscope slides; Superfrost Plus	Fisher Scientific	10149870
Microtome blades MX35 Premier (34°/80mm)	Fisher Scientific	3051835
RNase Away Solution	Fisher Scientific	10666421
RNALater Solution	ThermoFisher Scientific/Ambion Division	AM7021
Sterile Water, RNase-free	Baxter Healthcare	UKF7114
Fibrowax (pastillated)	VWR International	361427G
Cover Glass 22x64mm	VWR International	631-0880
DePex Mountant	VWR International	360294H
Ambion Ribo-Pure Blood kit	ThermoFisher Scientific/Ambion Division	*AM1928*

### Lead Contact and Materials Availability

Further information and requests for resources and reagents should be directed to and will be fulfilled by the Lead Contact, Costantino Pitzalis (c.pitzalis@qmul.ac.uk). This study did not generate new unique reagents.

### Experimental Model and Subject Details

#### Pathobiology of Early Arthritis Cohort (PEAC)

90 rheumatoid arthritis patients fulfilling 2010 ACR/EULAR RA Classification Criteria were enrolled at Barts Health NHS trust (London, UK) as part of the Medical Research Council (MRC) funded multi-center Pathobiology of Early Arthritis Cohort (PEAC). The study received ethical approval from the UK Health Research Authority (REC 05/Q0703/198, National Research Ethics Service Committee London – Dulwich). All patients gave written informed consent. Patients had clinically defined synovitis, but duration of symptoms of less than 12 months. Patient characteristics are summarized in Table S1. Exclusion criteria included all patients receiving corticosteroids, sDMARDs or biologic therapies. Upon enrolment and acquisition of demographic and clinical disease parameters, patients underwent minimally invasive ultrasound-guided synovial biopsy of a clinically active joint (see below).

### Method Details

#### Ultrasound-Guided Synovial Biopsy

We analyzed 90 synovial samples acquired through a minimally invasive US-guided synovial biopsy (Kelly et al., 2015) from patients presenting with early RA naive to therapy. Ultrasonography scores were collected at the time of biopsy for both the individual biopsied joint as well as a global joint score. Immediately prior to baseline US-guided synovial biopsy standard longitudinal images of the 1^st^-5^th^ metacarpo-phalangeal (MCP) joints and midline, radial, and ulnar views of both wrist joints were acquired in addition to standard images of the joint undergoing US-guided synovial biopsy as previously described (Kelly et al., 2015). Images subsequently underwent semiquantitative (SQ) assessment by a blinded assessor (IL) for both synovial thickening (ST) and power doppler activity (PD) according to standard EULAR-OMERACT US synovitis scores (grade 0-3) (Naredo et al., 2008). For each patient, baseline total mean (12max) ST (STUS) and PD (PDUS) scores were calculated by deriving the mean of the total scores for ST and PD for all 12 joints including maximal score in the wrist. STUS and PDUS were also recorded of the biopsied joint. All procedures were performed following written informed consent and were approved by the hospital’s ethics committee (REC 05/Q0703/198).

#### Clinical Assessments

At baseline clinical parameters including CRP, ESR, RF/ACPA positivity/titer and DAS28 were collected. Anonymized baseline radiographs of the hands and feet underwent scoring according to the modified Sharp van der Heijde scoring system by a trained reader.

#### Synovial Histology

3 μm paraffin embedded sections underwent standard H&E staining and then semiquantitative assessment for degree of synovitis according to a previously validated score (Krenn et al., 2006). In order to determine the degree of immune cell infiltration sequentially cut sections underwent staining for B cells (CD20), T cells (CD3), macrophages (CD68) and plasma cells (CD138) as previously reported (Humby et al., 2009), and mast cells (CD117). Sections underwent SQ scoring (0-4) for CD3, CD20, CD68 lining (CD68L) and sublining (CD68SL) and CD138 number (Humby et al., 2009). CD20+ aggregates within synovial tissue were graded (1-3) according to a scoring atlas as previously described (Manzo et al., 2005). Synovial biopsies were categorized into 3 separate synovial pathotypes according to the following criteria: i) Lympho-myeloid (L) presence of grade 2-3 CD20+ aggregates, (CD20 ≥ 2) and/or CD138 > 2; ii) Diffuse-Myeloid (M) CD68SL ≥ 2, CD20 ≤ 1 and/or CD3 ≥ 1, CD138 ≤ 2 and iii) pauci-immune Fibroid (F) CD68SL < 2 and CD3, CD20, CD138 < 1. Automated image analysis and cell counting (cellSens, Olympus) was used to calculate the density of CD117+ mast cells (number/mm^2^).

#### RNA Extraction Procedure

All tissue samples were maintained on ice and homogenized in a fume hood using a rotor-stator benchtop laboratory homogenizer. Samples were homogenized at short five-second intervals until all the tissue had been sheared/homogenized. The probe of the homogenizer was cleaned thoroughly in between samples by washing initially in RNase Away solution (Fisher Scientific, UK), followed by four washes in sterile/RNase-free water (Baxter Healthcare Ltd, UK). RNA was extracted from a minimum of 10mg of synovial tissue homogenized at 4°C in Trizol reagent (ThermoFisher Scientific, Invitrogen Division, UK). Chloroform was mixed with the lysate and following centrifugation the aqueous RNA layer was transferred to a new microcentrifuge tube. Isopropanol at 4°C was mixed with the RNA layer. Following incubation and centrifugation, the isopropanol was removed and the RNA pellet washed with 70% ethanol. The pellet was re-dissolved in RNase-free water.

Whole blood samples were preserved in RNALater solution (ThermoFisher Scientific, UK) (500μL whole blood: 1.3mL RNALater solution) and stored at −80°C prior to extraction. Blood samples in RNALater solution were thawed on ice and RNA prepared using the Ambion Ribo-Pure Blood kit (ThermoFisher Scientific, UK), as per the manufacturer’s instructions.

The concentration/purity of RNA samples was measured using the NanoDrop 2000C (Lab Tech, UK) and RNA quality (RIN) was assessed by Agilent 2100 Bioanalyser (Agilent Technologies, UK) and 2200 TapeStation (Agilent Technologies).

#### RNA Sequencing

Where available, 1 μg of total RNA was used as an input material for library preparation using TruSeq RNA Sample Preparation Kit v2 (Illumina). Generated libraries were amplified with 10 cycles of PCR. Size of the libraries was confirmed using 2200 TapeStation and High Sensitivity D1K screen tape (Agilent Technologies) and their concentration was determined by qPCR based method using Library quantification kit (KAPA). The libraries were first multiplexed (five per lane) and then sequenced on Illumina HiSeq2500 (Illumina) to generate 50 million of paired end 75 base pair reads (154 samples) or 30 million of single end 50 base pair reads (10 samples).

### Quantification and Statistical Analysis

#### RNA-Sequencing Data Processing

Transcript abundance was derived from paired (154 samples) or single (10 samples) FASTQ files over GENCODE v24/GRCh38 transcripts using Kallisto v0.43.0 (Bray et al., 2016). Transcript abundances and average transcript lengths were imported into R using Bioconductor package tximport 1.4.0 and summarized over NCBI RefSeq transcript isoforms. Imported abundances were normalized in R, including a correction for average transcript length and incorporating batch, sex, and pathotype as model covariates, using DESeq2 1.14.1 (Love et al., 2014). Transcript abundances underwent regularized log expression (RLE) transformation. Principal components analysis (PCA) was performed on the RLE normalized data and paired plots of first 10 eigenvectors were generated to identify outliers. After removal of three synovium RNA sample outliers, transcript abundances for the remaining synovium (n = 87) and blood (n = 67) samples were re-imported into R, normalized, and underwent RLE transformation followed by PCA again to confirm homogeneity of each dataset.

#### Identification of Cell-Specific Gene Sets

RLE normalized FANTOM5 data were downloaded from http://fantom.gsc.riken.jp/5/data/. Data were subsetted to include only unmanipulated and uncultured primary tissues (derived cells, stimulated cells, and cell lines were excluded) and restricted to NCBI gene transcripts. For each gene only the CAGE peak with the highest mean expression was used. Data were Z score normalized across all primary tissues and expression of each gene ranked across all tissues. A specificity score was determined for all genes by counting the number of tissues showing increased gene expression Z score >3 (i.e., more than 3 SD above the mean expression across all tissues), so that the most tissue specific genes would have the lowest specificity scores. After different cut-offs were tested for robustness, genes were considered specific to a tissue type using the following criteria: i) the level of gene expression in that tissue was in the top three tissues (i.e., rank 1-3); ii) Z score >5 (i.e., >5 SD above the mean expression across all tissues); iii) specificity score < 10 tissues. Gene modules for different cell types were consistent with lists of genes previously published by the FANTOM5 consortium for several cell types (Motakis et al., 2014, Schmidl et al., 2014).

#### Gene Module Scoring

Gene module scores for synovial RNA-seq data were derived by singular value decomposition (SVD) for each gene module matrix using methodology described in detail by other studies (Langfelder and Horvath, 2007). Module scores specific for B cells, T cell subsets, monocyte/macrophage subsets, plasma cells, and mast cells were analyzed for correlation against relevant histological markers in synovial tissue. Statistical comparison of groups was performed using one-way ANOVA and post hoc Bonferroni test.

#### Differential Expression Analysis

Differential Expression analysis based on negative binomial distribution using regression models of normalized count data was performed using DESeq2 using a likelihood ratio test to compare variation between pathotype groups in synovium and peripheral blood RNA-seq samples, followed by pairwise comparisons between Lympho-myeloid, Diffuse-Myeloid and pauci-immune Fibroid groups. P values were converted to Q values based on Benjamini-Hochberg false discovery rate (FDR), using FDR cut-off set at Q < 0.05 to define differentially expressed genes. MA and volcano plots were generated to illustrate the distribution of significant genes in each comparison.

#### 3D Volcano Plot

Three-way differential expression was visualized by a 3D cylindrical volcano plot using R package plotly 4.6.0. RLE counts were Z-score normalized and mean Z scores calculated for the three pathotype groups (L, M, F) for each gene. This three dimensional data were reduced to two dimensional polar coordinate system analogous to color space conversion of red, green, blue (RGB) color space to hue, saturation, value (HSV):

$$x=\;\frac{\sqrt{3}\;}{2}\;\left(M-F\right)$$

$$y=L-\frac{1}{2}\left(M+F\right)$$

ϑ=atan2(y,x)

$$r=\sqrt{x^{2}+y^{2}}$$

Thus Lympho-myeloid, Diffuse-Myeloid, pauci-immune Fibroid vectors were mapped to three axes in the horizontal plane (see polar plots, Figures 2B and 2C). Fold change can be used as an alternative to Z score for the radial scale without affecting θ. *z* axis shows –log_10_ p value for likelihood ratio test comparing all three groups. Genes with FDR-adjusted p value for likelihood ratio test < 0.05 were considered significant. Significant genes were color-coded based on pairwise statistical tests using the minimum group mean as reference. Genes which were significantly upregulated in one group alone were colored using primary colors, i.e., Lympho-myeloid, blue; Diffuse-Myeloid, red; and pauci-immune Fibroid, green. Genes upregulated in two groups compared to the minimum reference group were depicted using secondary colors i.e., genes upregulated in Lympho-myeloid and Diffuse-Myeloid compared to pauci-immune Fibroid: purple; upregulated in Diffuse-Myeloid and pauci-immune Fibroid versus Lympho-myeloid: yellow; upregulated in Lympho-myeloid and pauci-immune Fibroid versus Diffuse-Myeloid: cyan. Non-significant genes are colored gray.

#### Hierarchical Clustering

Hierarchical clustering on 2964 differentially expressed synovium genes (FDR < 0.05, log_2_ fold change >1) was performed using Euclidean distance metric and Ward’s linkage method and plotted using the ComplexHeatmap package 1.14.0 in R. Color tracks for histology data for CD3, CD20, CD68L/SL, CD138 and overall pathotype were included to aid interpretation.

#### Pathway Analysis

Pathway analysis was performed for each of four clusters of genes which were identified via hierarchical clustering by gene enrichment analysis using Ingenuity Pathway Analysis (IPA, QIAGEN, Redwood City, CA, USA). Upstream regulators were identified for pathotypes and gene clusters. All p values were FDR adjusted. Pathway analysis was undirected and detected enrichment against a background of all human genes.

#### Modular Gene Analysis

RNA-seq read counts were analyzed for differential gene module expression, using the Bioconductor package Quantitative Set Analysis for Gene Expression (QuSAGE, version 2.10.0) (Yaari et al., 2013), using gene modules derived from Li et al. (2014). Where stated, p values were corrected for multiple testing using Storey’s q value. Weighted correlation network analysis (WGCNA) on synovium RNA-seq was performed using R package WGCNA (Langfelder and Horvath, 2007). Gene modules were annotated against 13 cell types identified by single cell RNA-seq of RA synovium (Stephenson et al., 2018), using enrichment testing by hypergeometric test for module genes differentially upregulated in each single cell RNA-seq cell type. Modules were also annotated for pathways using REACTOME and Kegg databases.

### Data and Code Availability

The RNA-seq data have been deposited in ArrayExpress under Accession code E-MTAB-6141.

### Additional Resources

#### Web Interface

To facilitate data exploration, we developed a web interface available at https://peac.hpc.qmul.ac.uk/. The website was constructed using R shiny server 1.5.2 with interactive plots generated using R plotly 4.7.1. This allows interactive 3D visualization of the three-way volcano plot allowing users to click on individual genes to see their expression. A searchable interface is available to examine relationships between individual synovial and blood gene transcript levels and histological, clinical, and radiographic parameters, and clinical response at 6 months. A selectable table of synovial genes differentially expressed in different pathotypes is included. An interactive interface allows the gene module analysis to be explored for relationships between modules and clinical parameters. Figure S4 summarizes the main features of the website.

## Supplemental Information

Document S1. Figures S1–S7 and Tables S1– S3

Table S4. Cell-Lineage-Specific Gene Markers Derived from FANTOM5, Related to Figures 1B–1D, 2, and S1 See separate excel file filename Table S4.xlsx.

Table S5. Synovium Gene Modules Derived by Weighted Gene Correlation Network Analysis, Related to Figure 5C See separate csv file filename Table S5.csv.

Document S2. Article plus Supplemental Information
