# Supplementary material for: Molecular Portraits of Early Rheumatoid Arthritis Identify Clinical and Treatment Response Phenotypes
Source: Cell Rep. 2019 Aug 27;28(9):2455–2470.e5. doi: 10.1016/j.celrep.2019.07.091 (PMC6718830; doi:10.1016/j.celrep.2019.07.091)
Supplement: Document S1. Figures S1–S7 and Tables S1– S3 [file mmc1.pdf]

Cell Reports, Volume *28*

Supplemental Information

Molecular Portraits of Early Rheumatoid Arthritis Identify Clinical and Treatment Response Phenotypes

Myles J. Lewis, Michael R. Barnes, Kevin Blighe, Katriona Goldmann, Sharmila Rana, Jason A. Hackney, Nandhini Ramamoorthi, Christopher R. John, David S. Watson, Sarah K. Kummerfeld, Rebecca Hands, Sudeh Riahi, Vidalba Rocher-Ros, Felice Rivellese, Frances Humby, Stephen Kelly, Michele Bombardieri, Nora Ng, Maria DiCicco, Désirée van der Heijde, Robert Landewé, Annette van der Helm-van Mil, Alberto Cauli, Iain B. McInnes, Christopher D. Buckley, Ernest Choy, Peter C. Taylor, Michael J. Townsend, and Costantino Pitzalis

Supplemental Information

Molecular portraits of early rheumatoid arthritis identify clinical and treatment response phenotypes

Table S1. Baseline demographics of treatment naïve individuals with early rheumatoid arthritis, related to Figure 1

	**Whole cohort**	**Lympho-myeloid**	**Diffuse Myeloid**	**Pauci-immune fibroid**	**Ungraded**	**P value**
n	90	45	20	16	6	
Age (years)	54 ± 17	53 ± 2.4	51 ± 4.1	55 ± 3.9	47 ± 5.0	0.76
Sex female:male	64:23 (74%:26%)	34:11	14:6	12:4	4:2	0.94
Disease duration (months)	5.6 ± 3.2	6.3 ± 0.46	4.8 ± 0.79	6.1 ± 0.80	5.4 ± 1.4	0.25
RF positive (%)	62 (71%)	32 (71%)	14 (70%)	12 (75%)	3 (50%)	1.0
CCP positive (%)	64 (74%)	35 (78%)	13 (65%)	12 (75%)	4 (66%)	0.58
DAS28	5.8 ± 1.3	6.3 ± 0.19	5.55 ± 0.25	5.48 ± 0.41	6.40 ± 0.54	0.051
ESR (mm/hr)	44 ± 29	54 ± 4.4	36 ± 5.5	35 ± 7.8	33 ± 7.3	0.026
CRP (µg/mL)	23 ± 33	26 ± 4.0	17 ± 5.5	25 ± 13.8	7 ± 2.9	0.45
TJC	12 ± 7.6	13 ± 1.1	9.6 ± 1.4	12 ± 2.3	17 ± 3.3	0.20
SJC	7.9 ± 5.7	8.8 ± 0.87	6.8 ± 0.93	6.6 ± 1.7	9.2 ± 2.7	0.26
VAS	63 ± 24	67 ± 3.6	63 ± 5.0	59 ± 6.5	78 ± 6.5	0.56
HAQ	1.54 ± 0.76	1.63 ± 0.12	1.37 ± 0.12	1.59 ± 0.21	1.50 ± 0.39	0.31
US Synovial thickness (12max)	16.7 ± 9.1	17.0 ± 1.5	14.9 ± 1.9	17.7 ± 2.1	14.0 ± NA	0.67
US Power Doppler (12max)	7.0 ± 6.2	8.0 ± 0.99	5.9 ± 1.1	5.9 ± 1.6	3.0 ± NA	0.42
x-ray SvH score	3.7 ± 8.0	4.5 ± 1.5	1.5 ± 0.57	3.5 ± 1.3	3.0 ± 2.1	0.14

Data for whole cohort expressed as mean ± SD. Data for pathotype groups expressed as mean ± SEM. Statistical analysis for difference between pathotype groups (excluding ungraded) and continuous variables calculated by one-way ANOVA and categorical variables by Fisher exact test.

Abbreviations: RF: rheumatoid factor; CCP: cyclic citrullinated peptide antibody; DAS28: 28-joint disease activity score; ESR: erythrocyte sedimentation rate; CRP: C-reactive protein; TJC: tender joint count; SJC: swollen joint count; HAQ: health assessment questionnaire score; US: ultrasound; SvH: Sharp van der Heijde score.

Table S2. Distribution of synovial pathotypes, related to Figure 1

	Lymphoid	Myeloid	Fibroid	Ungraded
n (synovium RNA pre-QC)	46 (51%)	21 (23%)	17 (19%)	6 (7%)
n (synovium RNA post-QC)	45 (52%)	20 (23%)	16 (18%)	6 (7%)
n (blood RNA pre/post-QC)	30 (45%)	13 (19%)	16 (24%)	8 (12%)

Table S3. Demographics of disease modifying anti-rheumatic drug (DMARD) therapy at 6 months, related to Figures 6F-H & 7

MTX/SZP	65 (55%)
MTX/HCQ	22 (19%)
MTX/SZP/HCQ	8 (6.8%)
MTX alone	8 (6.8%)
HCQ alone	7 (5.9%)
SZP alone	4 (3.4%)
None	3 (2.5%)

Abbreviations: MTX: methotrexate; SZP: sulfasalazine; HCQ: hydroxychloroquine.

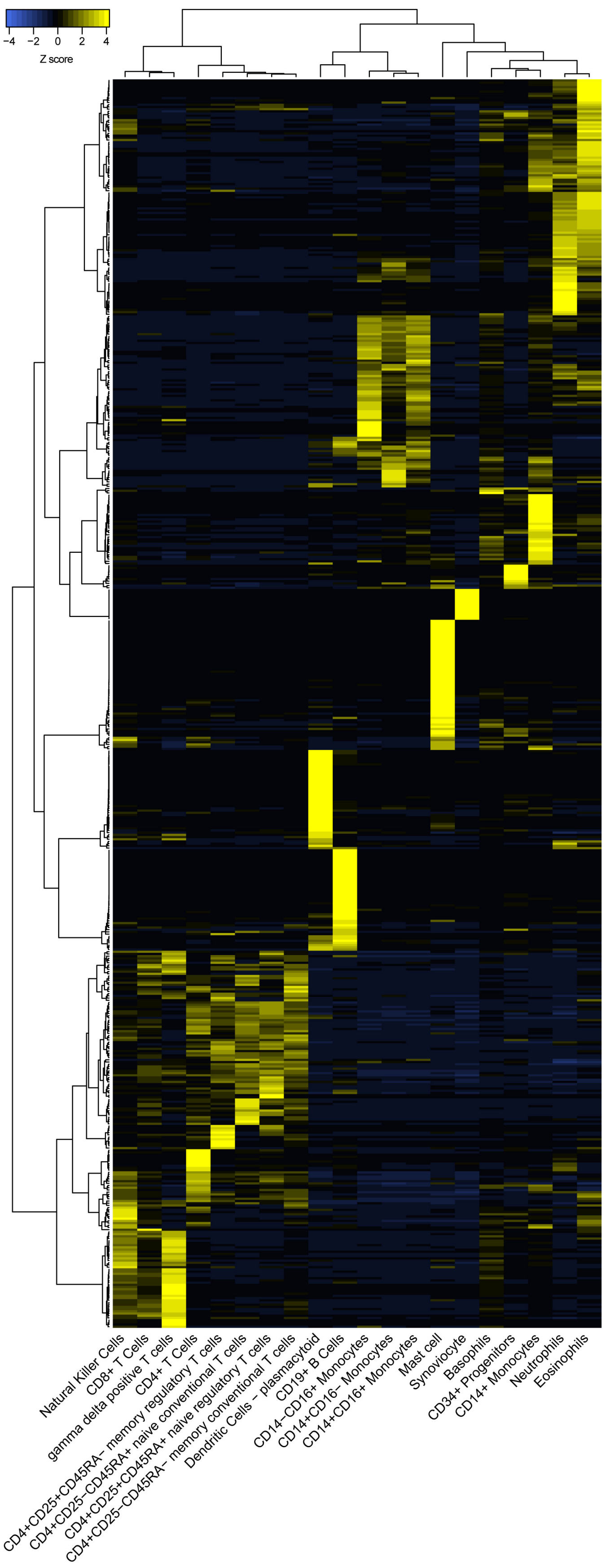

Figure S1. Heatmap of FANTOM5 CAGE sequencing gene expression for cell-specific immune cell genes, related to Figure 1 & 2

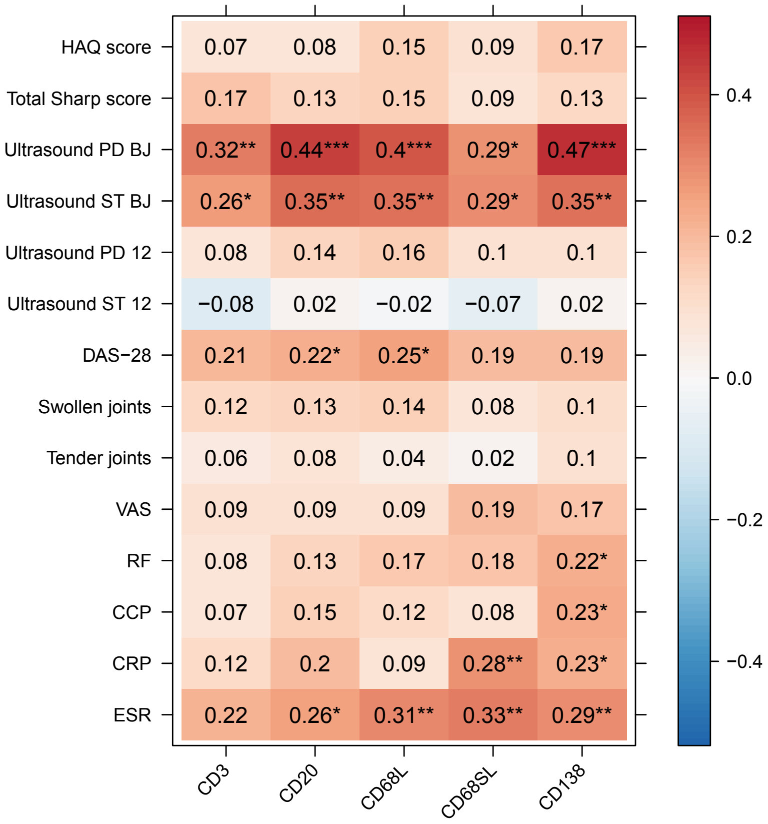

Figure S2. Correlation plot of clinical parameters against synovial histology scores, related to Figure 2

Numerical values represent Spearman rho values. *P<0.05, **P<0.01, ***P<0.001.

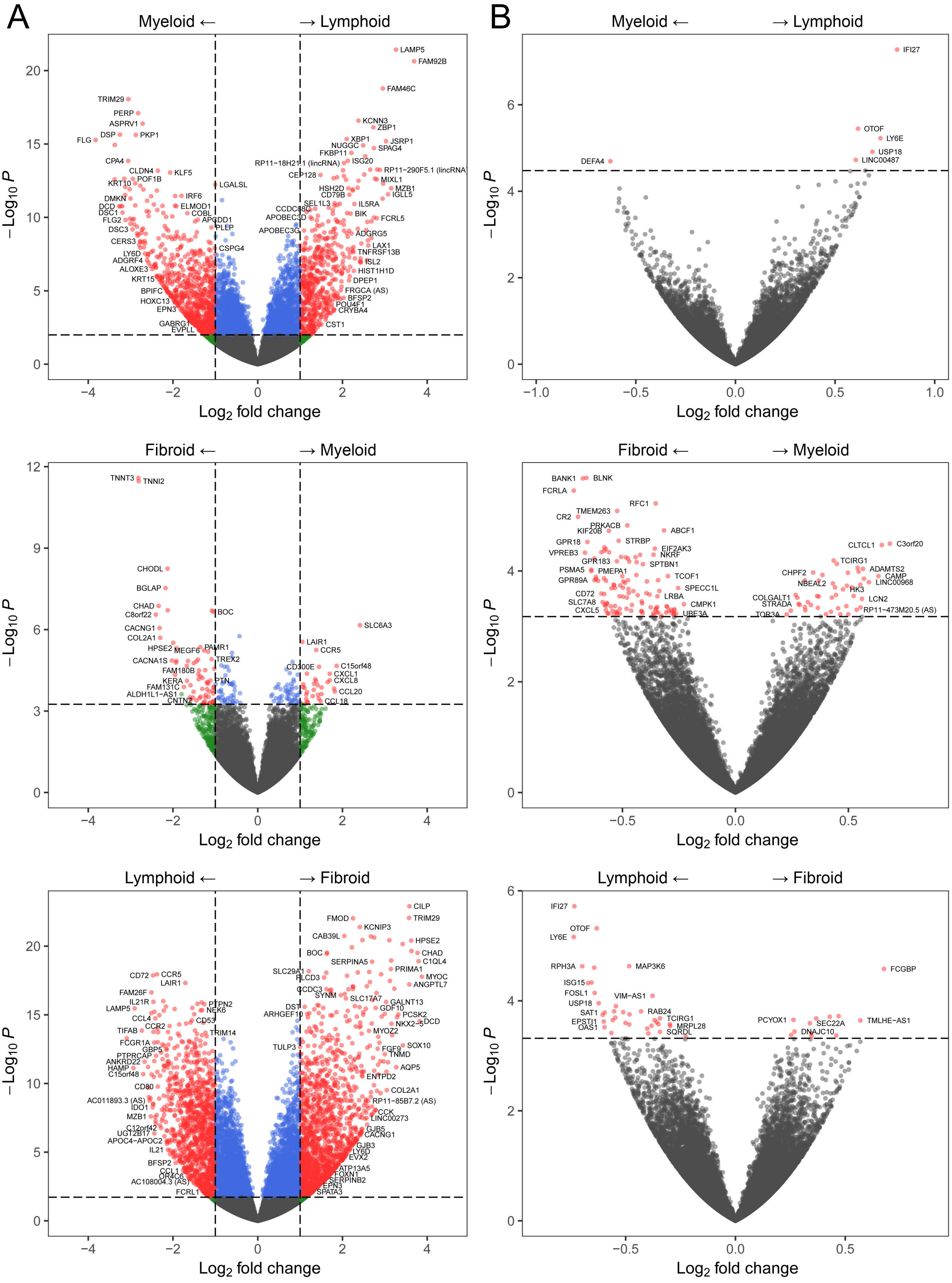

Figure S3. Conventional volcano plots of pairwise differential gene expression in rheumatoid arthritis RNA-sequencing of synovium and peripheral blood, related to Figure 3A,B
Volcano plots of pairwise comparisons of differentially expressed genes (red) between the three histological groups (Lymph-myeloid, Diffuse Myeloid and pauci-immune Fibroid) by RNA-sequencing of (A) synovial tissue or (B) whole blood in early rheumatoid arthritis. Statistical analysis by DESeq2 likelihood ratio test. Horizontal dotted line represents FDR<0.05.

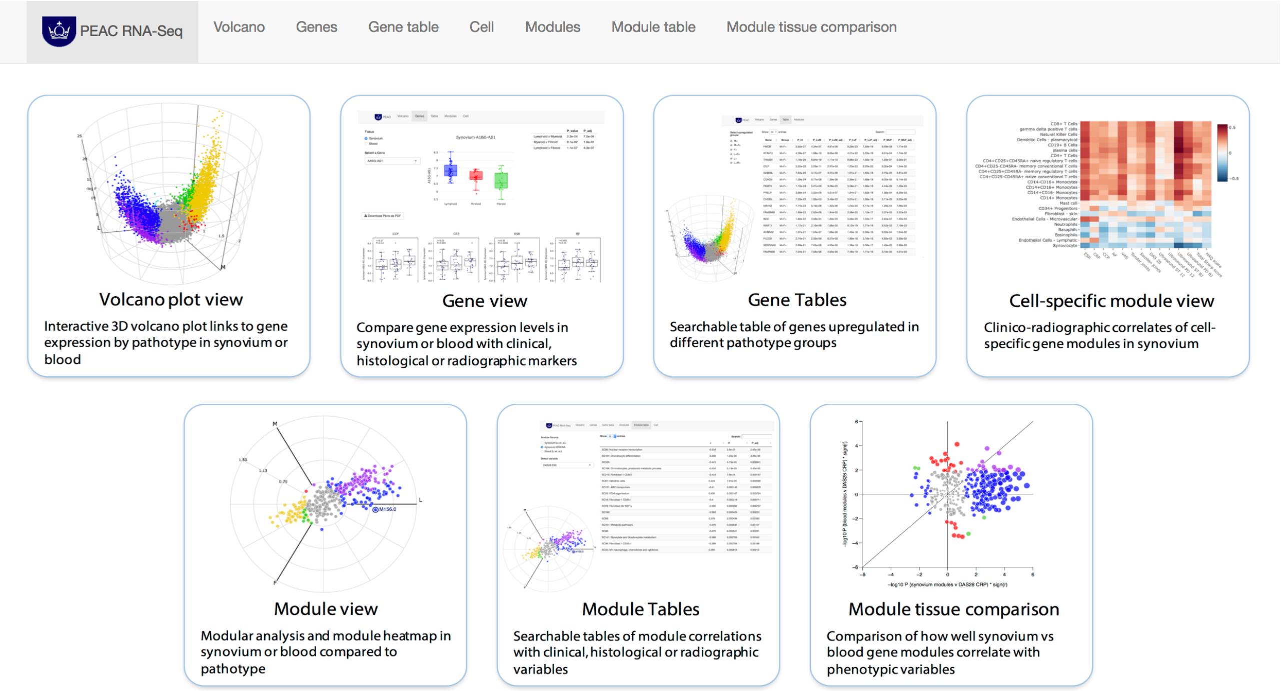

Figure S4. PEAC RNA-seq web interface, related to Figure 5 & 7

Summary of main features of PEAC RNA-seq web interface (http://peac.hpc.qmul.ac.uk/) allowing users to explore the data analysis using interactive plots and tables and searchable interface for genes and modules.

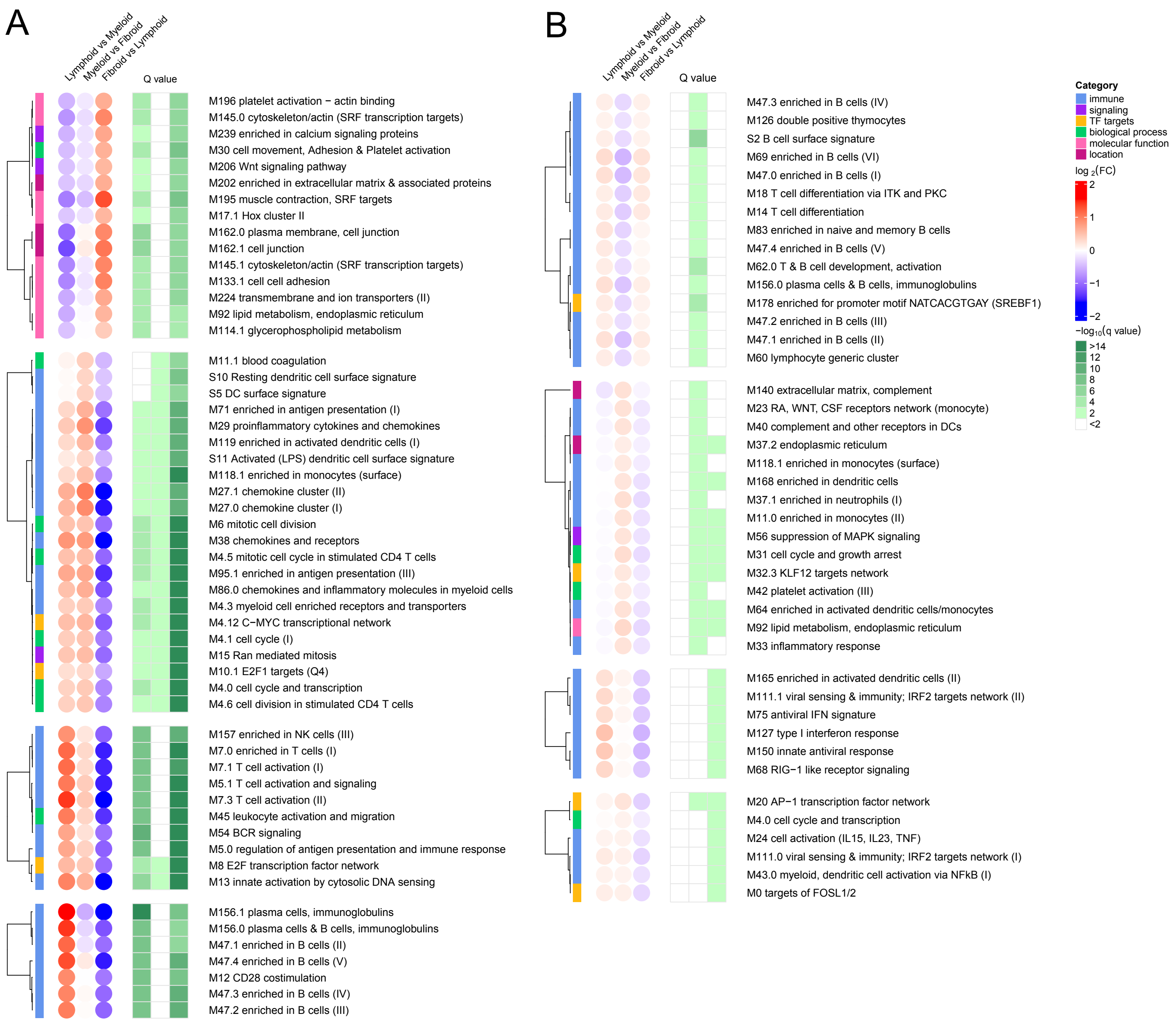

Figure S5. Modular analysis of synovial and blood RNA-sequencing, related to Figure 5A,B
Synovium and blood gene module plots based on blood microarray modules (Li et al, 2014), analysed using QuSAGE. Significant modules FDR-corrected P<0.01 are shown for paired comparisons of Lymphoid, Myeloid and Fibroid samples in (A) synovium and (B) peripheral blood. Top 15 most statistically significant modules are shown for each paired comparison. FDR-corrected P values shown by green colour scale.

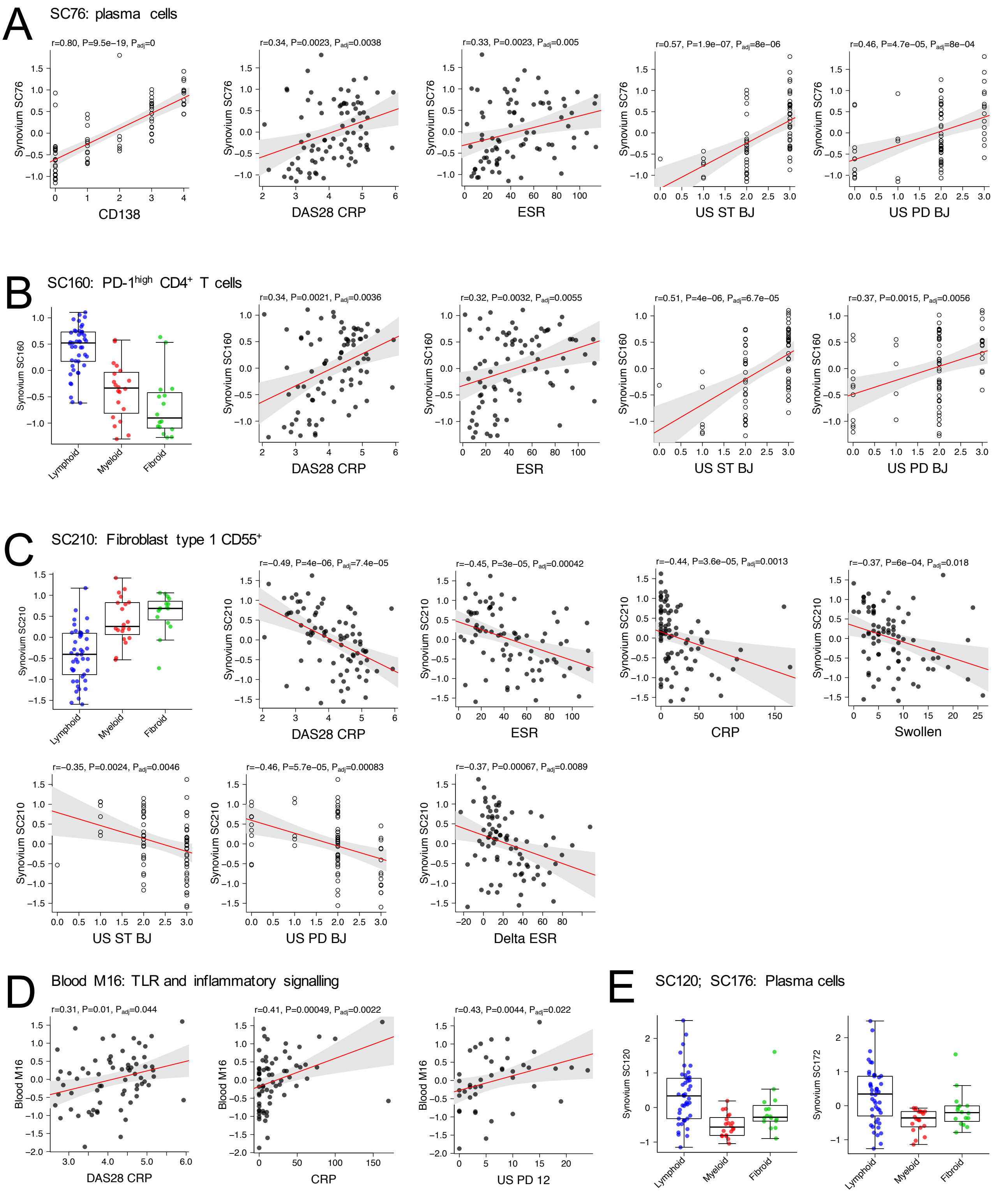

Figure S6. Correlation of histological, clinical and radiographic data with selected single cell RNA-seq annotated WGCNA modules and blood modules, related to Figure 5C

See website (http://peac.hpc.qmul.ac.uk/) to browse full correlation data for all modules and all variables, under the Modules tab.

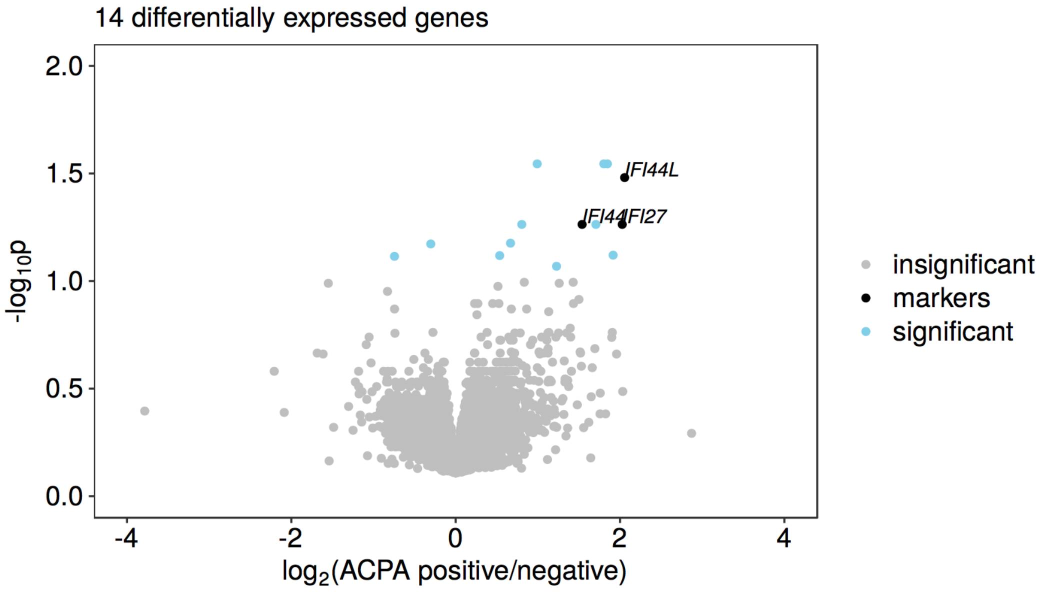

Figure S7. Blood RNA-seq differentially gene expression in anti-CCP antibody positive versus negative RA individuals, related to Figure 6A

Differentially expressed genes in blood RNA-seq showing upregulated interferon response genes in anti-CCP antibody (ACPA) positive RA individuals compared to ACPA negative individuals at FDR<0.10.
